# Stability and Robustness of a Generalized Pump-Leak Model for Epithelial Vesicles

**DOI:** 10.1007/s11538-026-01683-7

**Published:** 2026-07-14

**Authors:** Kerry Tarrant, Alan R. Kay, Zahra Aminzare

**Affiliations:** 1 Department of Mathematics, University of Iowa, Iowa City, IA USA; 2 Department of Biology, University of Iowa, Iowa City, IA USA

**Keywords:** Pump-leak model, Volume stabilization, Sensitivity analysis, Robustness

## Abstract

Epithelial cells stabilize ion concentrations and volume through coordinated membrane pumps, ion channels, and paracellular pathways, which can be modeled by classical single-compartment pump-leak equations (PLEs). Many epithelial functions, however, depend on the interaction between a cell and an enclosed luminal space, a geometry that cannot be captured by classical PLEs. To address this, we develop a two-compartment model consisting of an intracellular compartment coupled to a luminal compartment through the apical membrane, with both compartments interfacing an infinite extracellular bath and connected to it through the basolateral membrane and a paracellular pathway. Building on the five-dimensional single-cell PLEs, we formulate a ten-dimensional PLE system for this geometry and derive analytical equilibria and steady-state formulas for both the passive system and the Na^+^/K^+^-ATPase (NKA) driven active system. We characterize how these states depend on physiologically relevant parameters, analyze local stability across wide parameter ranges, and apply global sensitivity and robustness methods to identify the principal determinants of ion and volume homeostasis. Our focus is on closed epithelial systems in which the lumen volume relaxes to a steady state, rather than on fluid-secreting epithelia with time-varying luminal volume. We quantify apical and basolateral membrane potentials at steady state; the basolateral potential varies widely across parameter regimes, whereas the apical potential remains comparatively small in magnitude. The model reveals fundamental differences between basolateral and apical placement of the NKA, including the onset of luminal volume expansion when apical potassium recycling is insufficient. More broadly, this framework provides a mathematically tractable and physiologically grounded foundation for studying epithelial transport and for predicting conditions under which pump localization and conductance changes lead to stable function or pathological lumen expansion.

## Introduction

All live cells function under the constant threat of inundation by osmotic water fluxes that are driven by impermeant molecules within cells. This so-called *Donnan effect* is generated because cells have membranes that are permeable to water and contain impermeant molecules, such as metabolites, which ensures that water will flow continuously into the cell unless countermeasures are taken (Sperelakis 2012). Eukaryotes employ a variety of mechanisms to counter the Donnan effect. Plant cells have rigid cell walls that constrain swelling and allow them to sustain a turgor pressure (Höfte and Voxeur 2017) that opposes the osmotic influx of water. In contrast animal cells pump $\hbox {Na}^+$ ions out, which establishes a dynamic steady state, where the intracellular osmolarity balances that of the extracellular osmolarity. This pump-leak mechanism (PLM) also ensures that there is osmotic “room” in the cell to accommodate about 100 mM of a variety of metabolites, which are needed for the cell to survive (Kay and Blaustein 2019; Tosteson and Hoffman 1960). In all animal cells the sodium pump is the Na^+^/K^+^-ATPase (NKA), which transports three $\hbox {Na}^+$ out of the cell for every two $\hbox {K}^+$ pumped in, at the expense of one ATP molecule (Fedosova et al. 2021).

Much work has been done to characterize the behavior of the single cell PLM, showing that it establishes a robust means for stabilizing volume, as well as accommodating metabolites by moving chloride out of the cell, as well as establishing a negative membrane potential. The PLM is described by five algebraic-differential equations–collectively known as the pump-leak equations (PLEs)–that model the time-dependent changes in ionic concentrations, cell volume, and voltage. These equations have been rigorously analyzed for the single-cell setting (Hoppensteadt and Peskin 2002; Fraser and Huang 2007; Keener and Sneyd 2009; Mori 2012). In this case, the cell is bathed in an infinite, well-mixed extracellular medium and exchanges ions and water across a single membrane, and more recent descriptions and mathematical inquiries have incorporated cation-chloride cotransporters (Aminzare and Kay 2024). The PLEs create a system that ensures the emergence of a steady state without relying on a narrowly tuned parameter range.

**Fig. 1:**
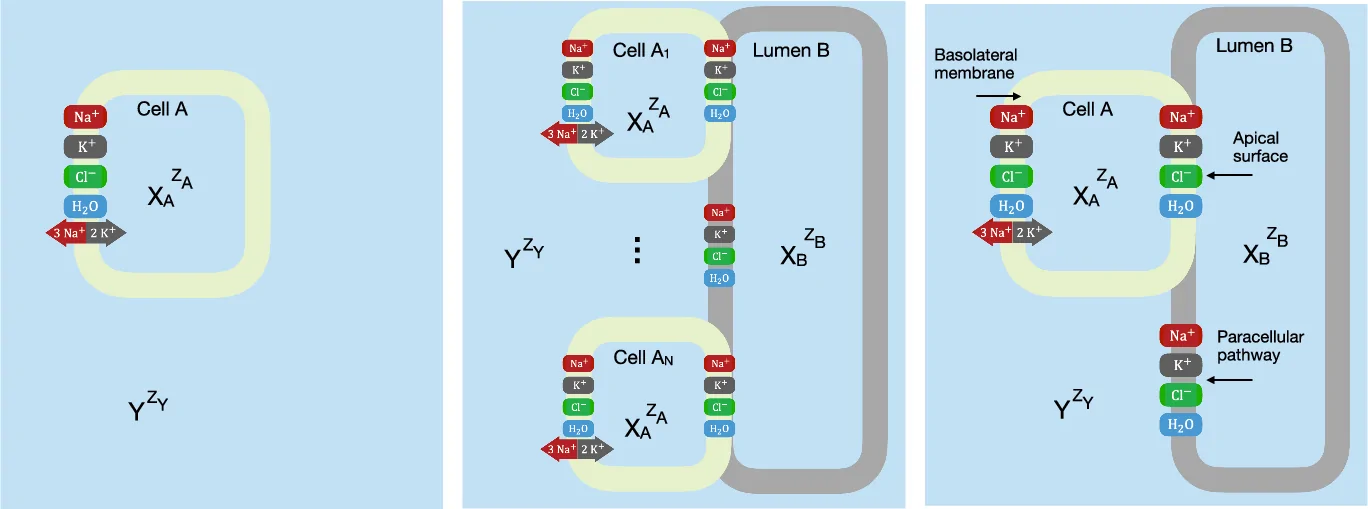
(Left) Schematic of a single-cell featuring Na^+^, K^+^, Cl^-^, and water channels; the Na^+^/K^+^-ATPase (NKA) pump; and impermeant molecules inside (X) and outside (Y) the cell. (Middle) Schematic of epithelial vesicle $A_1\cdots A_NBp$, a layer of *N* epithelial cells $A_1,\cdots , A_N$ surrounds a lumen B, which contains, water, ions and small metabolites. In this structure, cells connect to the lumen via the apical surface and to the extracellular fluid via the basolateral membrane. The lumen B is also connected to the extracellular fluid through a paracellular pathway (p in $A_1\cdots A_NBp$ system refers to the paracellular pathway). (Right) Schematic of the ABp model, a simplified version of $A_1\cdots A_NBp$ where the *N* cells collapsed to a single cell (color figure online)

**Epithelia** are ubiquitous in animals, often providing the working end of an organ, whose function is to actively transport ions, molecules and water, or to serve as a barrier (Boron and Boulpaep 2016). Simple epithelia consist of cells arranged in a tiled monolayer (Caceres et al. 2017). Junctions between adjacent epithelial cells are formed by transmembrane proteins that connect adjacent cells. Physiologists classify epithelia as either “tight” or “leaky,” which is determined by the permeability characteristics of these intercellular junctions (Sackin and Palmer 2013). Epithelia exhibit structural and functional asymmetry, with distinct populations of ion channels and transporters distributed between the apical membrane (facing the lumen) and the basolateral membrane (facing the interstitial space). This asymmetric distribution enables epithelia to transport ions and water vectorially.

Our objective is to model a rather specialized epithelium, which we term an “epithelial vesicle.” In these structures a layer of epithelial cells surrounds a lumen, which contains, water, ions and small metabolites (Figure 1, middle). The following are examples of epithelial vesicles: the ventricular system in the brain (Kandel et al. 2013), the scala media in the cochlea (Patuzzi 2011), follicles of the thyroid, ovarian follicles, and embryonic structures like, the blastula, and the otic vesicles (Wolpert et al. 2015). In all these cases the size of the lumen remains constant, at least in the short term. If all cells in the epithelial vesicle are the same, and the cells and lumen are small (i.e. of the order of a few microns), the cells can be collapsed into a single compartment A (Figure 1, right), since diffusion is rapid over short distances (Berg 1993). We will term this a static ABp system, where the volumes of A and B reach a constant steady state. This contrasts with what happens in a transporting epithelial system where the volume of B continuously grows as a fluid is secreted in it, see e.g., Palk et al. (2010); Sharp et al. (2015); Vera-Sigüenza et al. (2018).

We develop a two-component PLM in which compartment A represents an epithelial cell bordering the infinite extracellular interstitial fluid (ISF), and compartment B represents the lumen. Furthermore, the lumen can be coupled to the extracellular space by a paracellular pathway (p), hence the model is termed the ABp model. The permeability of the paracellular pathway is determined by occludin and claudin molecules which span the gap between epithelial cells (Tsukita et al. 2019). Claudin molecules (23 genes in humans), can establish ion selective pathways in the space between epithelial cells. Claudins allow the selective permeation of ions and water, depending on the claudin gene expressed in the epithelium, or as a barrier to ion or water fluxes.

In the absence of a paracellular pathway, the ABp model reduces to an AB system, where compartment B is contained within A (Figure 7). The AB system can serve as a model for intracellular organelles, like lysosomes, nuclei, endoplasmic reticulum, etc. Similar models have been developed to simulate various kinds of epithelial transport (Larsen et al. 2000; Nickerson et al. 2020; Weinstein 1992; Latta et al. 1984; Lew et al. 1979; Weinstein and Stephenson 1981), however crucially in these models the compartments flanking the epithelium have been assumed to be infinite and have fixed concentrations. In contrast, here we allow the concentrations of ions in B to vary, as well as its volume and voltage.

In the static ABp model, where in the steady state the volumes of all compartments reach a stable volume, the fluxes of any ion or water into and out of a compartment must be balanced. We will show that it is possible for there to be persistent cyclic flows of ions through the model, which do not need to be balanced.

In this work we focus on what we term “cell volume stabilization,” which is provided by the PLM through the action of the NKA. This is in contrast to what physiologists refer to as cell volume regulation (CVR) that encompasses transport processes that are recruited when the extracellular osmolarity is changed (Hoffmann et al. 2009).

Many physiological models of epithelial transport include several transporters and channels in the attempt to mimic the behavior of a particular system, but they seldom attempt to dissect the mechanisms at play. It is noteworthy that many do not seem to be aware of the role that the sodium pump plays in volume stabilization. This paper is part of a project to systematically explore the theoretical underpinnings of epithelial transport rather than an attempt to model a specific system. This project is undertaken in the spirit of the mathematical biosciences, as an attempt to lay bare the core of the operation of a system (Cohen 2004). Such ventures have sometimes revealed unexpected properties of the system beyond the reach of intuition (Guttman et al. 1980).

Understanding the collective behavior of these multicellular arrangements is essential for explaining coordinated volume regulation, fluid secretion, and absorption, and may reveal emergent properties that do not appear in single-cell models.

Our main contributions to this work are as follows.

**Development of a mathematical framework.** We generalize the classical five-dimensional pump-leak equations for a single cell to a ten-dimensional system describing the coupled dynamics of ion concentrations, volume, and membrane potential in a two-compartment ABp system.

**Existence and local stability of steady states.** We derive parameter conditions guaranteeing the existence of equilibria and steady states, obtain explicit analytical expressions for these states, and establish their local stability.

**Robustness and sensitivity analysis in high-dimensional parameter space.** The ABp model depends on a large number of parameters. To assess robustness of the steady states and long-term behavior, we analyze model sensitivity across a broad parameter range using Latin hypercube sampling combined with Sobol variance-based sensitivity indices. This approach quantifies how uncertainty in individual parameters and their interactions contributes to variability in key model outputs.

**Analysis of pump localization.** We analyze two distinct pump configurations—placement on the basolateral membrane versus the apical surface—and characterize their effects on the existence and stability of steady states.

Our analysis leads to the following outcomes:

**1.** As in the classical single-cell pump–leak model, the $\hbox {Na}^+$/$\hbox {K}^+$-ATPase plays a central role in stabilizing the two-compartment system and regulating cell and lumen volume. Moreover, we show that the qualitative behavior of the ABp system is insensitive to the precise mathematical form of the pump, justifying the use of a constant pump rate to obtain tractable analytical results.

**2.** Global sensitivity analysis reveals a pronounced low-dimensional structure in parameter space. In particular, sodium conductances, extracellular sodium concentration, and, in some regimes, temperature account for a dominant fraction of output variance and can partially compensate for reduced pump activity, thereby shifting volume steady states.

**3.** Although potassium and chloride conductances are necessary for physiological operation of the ABp system, the steady-state behavior is comparatively insensitive to variations in these parameters over wide ranges.

**4.** Pump localization has qualitatively distinct consequences: while basolateral pump placement supports stable regulation of both compartments, apical pump placement leads to divergence of the luminal volume as the pump rate slightly increases.

The structure of the paper is as follows. Section 2 introduces the coupled pump-leak equations for a general two-compartment system. Section 3 derives analytical expressions for the equilibria of the purely passive system, in which ionic fluxes driven by electrochemical gradients are the sole transport mechanisms. Section 4 extends this analysis by incorporating active transport via the NKA pump on the basolateral membrane and establishes the corresponding steady-state solutions. Section 5 employs sensitivity analysis and Sobol indices to investigate the robustness of the coupled PLEs under parameter fluctuations and to identify mechanisms that promote volume regulation. Section 6 considers the complementary case in which the NKA pump is located on the apical surface, yielding steady-state expressions that depend explicitly on membrane conductances and the pump rate. We conclude in Section 7. All tables and numerical details are provided in the Appendix.

## Pump-Leak Equations for Two-Compartment Systems

The classical pump-leak equations (PLEs) comprise a system of four differential equations and one algebraic constraint describing the intracellular concentrations of $\hbox {Na}^+$, $\hbox {K}^+$, and $\hbox {Cl}^-$, together with cellular volume and membrane potential for a single cell immersed in an infinite bath. Introduced by Tosteson and Hoffman in 1960 (Tosteson and Hoffman 1960), this framework was subsequently extended to incorporate mechanisms of cell volume regulation (Jakobsson 1980) and epithelial transport (Lew et al. 1979). Mori (2012) later established the existence and uniqueness of an asymptotically stable steady state, followed by additional analytical developments (Keener and Sneyd 2009; Aminzare and Kay 2024).

In this section, we generalize the PLE framework to describe ionic transport, compartmental volumes, and membrane potentials in two-compartment systems, with particular emphasis on ABp models (illustrated in the right panel of Figure 1). The resulting formulation consists of ten coupled differential-algebraic equations, which we refer to as the *coupled PLEs*.

To accommodate the additional compartment, we introduce notations that distinguish quantities in the cytoplasm (compartment *A*), lumen (compartment *B*, which may itself be an extracellular space), and extracellular space (ISF). Variables and parameters associated with compartments *A* and *B* carry the subscripts _*A*_ and _*B*_, respectively, while extracellular quantities carry the subscript _*e*_. We further use the subscripts _*eA*_, _*AB*_, and _*eB*_ to identify the basolateral membrane (between *A* and extracellular ISF), the apical surface (between *A* and *B*), and the paracellular pathway (between *B* and extracellular ISF).

**Volume.** We assume that the solution in each compartment is composed entirely of water, allowing us to treat osmolarity and osmolality as equivalent measures of total solute concentration (Boron and Boulpaep 2016). Since the membrane is permeable to water, if there are differences in osmolarity across it, water will move by osmosis (Manning and Kay 2023). The osmolarities of the extra- and intracellular solutions are respectively $\mathcal {O}_e$ and $\mathcal {O}_j$ (for j=A,B):

1a $$\begin{aligned}&\mathcal {O}_e= [Na]_e+ [K]_e+ [Cl]_e+ [Y]_e,\end{aligned}$$

1b $$\begin{aligned}&\mathcal {O}_j= [Na]_j+ [K]_j+ [Cl]_j+ [X]_j, \end{aligned}$$

where $[\text {ion}]_e$ and $[\text {ion}]_j$ represent the extracellular concentrations and intracellular concentrations for compartment *j* (j=A,B), respectively. $[X]_j$ describes the concentration of impermeant molecules inside compartment *j* with average charge $z_{j}$ which include metabolites and macromolecules. Similarly, $[Y]_e$ with average charge $z_{Y}$ represents the extracellular impermeant molecules. Because osmosis is a colligative effect, one need only consider the number of moles of these molecules. Note that we assume $[Y]_e$ is constant and the number of moles of the intracellular impermeant molecule is constant, i.e., $X_j$ is constant. If $w_j$ is the compartment volume then $[X]_j={X_j}/{w_j}.$ We assume that the osmotic coefficient of all the ions is unity. This does not affect the qualitative behavior of the system, only rescale the final values of the steady states.

The Starling equation governs water flux in the system (Garcia et al. 2013; Boron and Boulpaep 2016; Manning and Kay 2023), which describes changes in volume as proportional to the osmolarity differences across membranes–the Starling equation normally includes the transmembrane pressure, which we omit since we assume that the compartments can stretch freely without developing tension and, thus, any pressure. The following equations express the change in volumes in compartments *A* and *B*:

2a $$\begin{aligned}&\frac{ d w_A}{ dt } = \underbrace{{\nu _{eA}} (\mathcal {O}_A-\mathcal {O}_e)}_{\text {baso. mem.}} + \underbrace{{\nu _{AB}} (\mathcal {O}_A-\mathcal {O}_B)}_{\text {ap. sur.}} \end{aligned}$$

2b $$\begin{aligned}&\frac{ d w_B}{ dt } = \underbrace{{\nu _{eB}}(\mathcal {O}_B-\mathcal {O}_e)}_{\text {para. pathway}} - \underbrace{{\nu _{AB}} (\mathcal {O}_A-\mathcal {O}_B)}_{\text {ap. sur.}} \end{aligned}$$

where the parameters $\nu _{eA}$, $\nu _{AB}$, and $\nu _{eB}$ are the osmotic permeability coefficients times the molar volume of water.

**Voltage.** Throughout the paper, the extracellular voltage serves as the reference potential and is fixed at zero, i.e., $V_e = 0$. For $j \in \{A,B\}$, the membrane potential in each compartment, measured relative to the extracellular potential, is modeled by the following algebraic equation derived from the definition of capacitance (Fraser and Huang 2007):

3 $$\begin{aligned} V_j= \frac{F w_j}{C_{m,j}} \left( [Na]_j+ [K]_j-[Cl]_j+ z_j [X]_j\right) , \end{aligned}$$

where *F* is Faraday’s constant and $C_{m,j}$ is the total compartment capacitance.

Basolateral and apical membrane potentials then follow from the differences in compartment potentials, i.e., $V_{eA}:= V_A-V_e=V_A-0=V_A$ and $V_{AB}:= V_A-V_B.$ We aim to choose parameters such that, at steady state, $V_{AB}\ne V_{eA}$, i.e., $V_B\ne 0$.

**Ion concentrations.** In the presence of an electrochemical gradient, ionic channels facilitate the passive transport of ions by allowing them to move freely down their electrochemical gradients, thereby reducing the difference in charge and concentration across the membrane. Channels act as selective gateways for specific ionic species, such as sodium, potassium, and chloride. Passive ionic currents through these channels are described using Ohm’s law, chosen because it is a reasonable approximation for how conductances actually behave. Additionally, its linear dependence on electrochemical potential makes subsequent analyses more tractable. Ohm’s law for passive ionic current is given by $i_{\text {ion}} = - z_{\text {ion}} {g}_{\text {ion},(\cdot )}(V_j-E_{\text {ion},j}- V_e),$ where $z_{\text {ion}}$ is the ionic valence, ${g}_{\text {ion},(\cdot )}$ is the channel conductance on a given membrane or pathway, $V_j$ is the membrane potential relative to the extracellular potential $V_e$, and $V_j- E_{\text {ion},j}- V_e$ is the driving force. The Nernst potential $E_{\text {ion},j}$ is

4 $$\begin{aligned} E_{\text {ion},j}= \frac{RT}{z_{\text {ion}}F} \ln \left( \frac{[\text {ion}]_e}{[\text {ion}]_j}\right) , \end{aligned}$$

where *R* is the universal gas constant and *T* is the absolute temperature. When $V_j- E_{\text {ion},j}> V_e$, cations (anions) tend to move out of (into) the compartment.

The net ionic flux into each compartment results from the combined effects of passive transport, described by Ohm’s law, and active transport processes localized to the membrane of compartment *A*. The equations governing the concentrations of Na^+^, K^+^, and Cl^-^ in compartment *A* are given by the following equations.

5a $$\begin{aligned} F\frac{d \left( w_A[Na]_A\right) }{dt}&= -g_{Na,eA}\left( V_A- E_{Na,A}\right) +{{\textbf {p}}}_{{{\textbf {Na,eA}}}} \end{aligned}$$

5b $$\begin{aligned}&\quad + {{\textbf {p}}}_{{{\textbf {Na,AB}}}}+ g_{Na,AB}\left( (V_B- E_{Na,B})-(V_A- E_{Na,A})\right) , \nonumber \\ F\frac{d(w_A[K]_A)}{dt}&= -g_{K,eA}\left( V_A- E_{K,A}\right) + {{\textbf {p}}}_{{{\textbf {K,eA}}}} \end{aligned}$$

5c $$\begin{aligned}&\quad + {{\textbf {p}}}_{{{\textbf {K,AB}}}}+ g_{K,AB}\left( (V_B- E_{K,B})-(V_A- E_{K,A})\right) , \nonumber \\ F\frac{d(w_A[Cl]_A)}{dt}&= g_{Cl,eA}\left( V_A- E_{Cl,A}\right) - g_{Cl,AB}\left( (V_B- E_{Cl,B})-(V_A- E_{Cl,A})\right) . \end{aligned}$$

Similarly, the equations for the ionic concentrations in compartment *B* are

6a $$\begin{aligned} F\frac{d \left( w_B[Na]_B\right) }{dt}&= -g_{Na,eB}\left( V_B- E_{Na,B}\right) \end{aligned}$$

6b $$\begin{aligned}&\quad - {{\textbf {p}}}_{{{\textbf {Na,AB}}}}+ g_{Na,AB}\left( (V_A- E_{Na,A})-(V_B- E_{Na,B})\right) , \nonumber \\ F\frac{d(w_B[K]_B)}{dt}&= -g_{K,eB}\left( V_B- E_{K,B}\right) \end{aligned}$$

6c $$\begin{aligned}&\quad - {{\textbf {p}}}_{{{\textbf {K,AB}}}}+ g_{K,AB}\left( (V_A- E_{K,A})-(V_B- E_{K,B})\right) , \nonumber \\ F\frac{d(w_B[Cl]_B)}{dt}&= g_{Cl,eB}\left( V_B- E_{Cl,B}\right) - g_{Cl,AB}\left( (V_A- E_{Cl,A})-(V_B- E_{Cl,B})\right) . \end{aligned}$$

In Equations (5)–(6), $g_{\text {ion},eA}$, $g_{\text {ion},eB}$, and $g_{\text {ion},AB}$ denote the conductance parameters along the basolateral membrane (between extracellular *e* and compartment *A*), paracellular pathway (between compartment *B* and extracellular *e*), and apical membrane (between compartments *A* and *B*), respectively.

Note that in these equations, we did not include the effective of the convective flux of water, which could result in solvent drag, as is believed to occur in the proximal tubule of the nephron (Weinstein 1992)

Active flux is given by the bold terms ${\textbf {p}}_\text {ion,eA}$ and ${\textbf {p}}_\text {ion,AB}$. We will define the active transport terms at the beginning of subsequent sections. The last term in each equation is the passive flux across the apical surface.

** Electroneutrality.** The total concentration of charge inside the compartments and in the extracellular space is given by:

$$\begin{aligned} Q_j&=[Na]_j+[K]_j-[Cl]_j+z_{j}[X]_j,\\ Q_e&=[Na]_e+[K]_e- [Cl]_e+ z_Y[Y]_e. \end{aligned}$$

Because of the energetic cost of separating charges, isolated solutions will have a net charge of zero, so we can set $Q_e=0$.

Following (Mori 2012, Equation (2.12)), the non-dimensionalization of Equation (3) becomes $\epsilon _j {\hat{V}}_j = {\hat{w}}_j \hat{Q}_j$ where $\hat{\cdot }$ denotes the non-dimensional variables and the constant $\epsilon _j$ is a non-dimensional parameter defined by $\epsilon _j = \frac{C_{m,j}RT}{F^2X_j}$ with values $\epsilon _A\approx 3\times 10^{-4}$ and $\epsilon _B\approx 3\times 10^{-5}$. Since $\epsilon _j$ is very small, ${\hat{w}}_j \hat{Q}_j$ becomes very small, too. Therefore, once we assume $Q_e=0$, we can conclude the so called “electroneutrality condition”

7 $$\begin{aligned} Q_j \;\sim \; 0. \end{aligned}$$

While electroneutrality approximates the net concentration of charges, voltages need not be zero (Keener and Sneyd 2009; Østby et al. 2009; Aminzare and Kay 2024). In the following sections, we will use electroneutrality condition (7) to explicitly derive the equilibrium and steady state values of the PLEs.

To maintain a constant extracellular osmolarity $\mathcal {O}_e$, we fix the values of $\mathcal {O}_e$, $[K]_e$, $[Y]_e$, and $z_{Y}$, and allow the concentrations $[Cl]_e$ and $[Na]_e$ to be determined by the following relationships. Note that all these quantities are held constant; this formulation simply enforces the constraint of constant extracellular osmolarity.

8a $$\begin{aligned}&[Cl]_e= \frac{1}{2}\left( \mathcal {O}_e+ \left( z_{Y}- 1\right) [Y]_e\right) , \end{aligned}$$

8b $$\begin{aligned}&[Na]_e= -[K]_e+[Cl]_e- z_{Y}[Y]_e. \end{aligned}$$

Equation (8a) is derived by combining the extracellular osmolarity in Equation (1a) and the electroneutrality Equation (7). Equation (8b) comes directly from the assumption of electroneutrality. Table 2 gives the default values of solute concentrations.

From this point forward, we refer to the system consisting of the volume equation (2), the voltage relation (3), and the ion concentration equations (5)–(6) collectively as the coupled PLEs for a general ABp system.

The right illustration in Figure 1 has some limitations. First, the volumes of compartments *A* and *B* are not drawn to scale; for a more precise representation, compartment *B* should be at least ten times larger than *A*. Second, the paracellular pathway is a narrow intercellular space through which ions and water flow. Despite these simplifications, the schematic highlights some features that will be useful later.

Default parameter values, sometimes called nominal values, are provided in Section 1. Tables 1-4 provide default values and descriptions for the parameters used in our model. Table 1 specifies the surface area for the basolateral and apical membranes, which are assumed to be identical unless otherwise stated, and the paracellular pathway. The product of area and conductance determines the effective number of channels in each membrane. Surface area calculates the distribution of leak channels and active transporters along surfaces and pathways. We assume that the number of channels and transporters does not change as the volume of the compartment changes. Area for the basolateral membrane, apical surface, and paracellular pathway will be denoted with $a_{eA}$, $a_{AB}$, and $a_{eB}$, respectively.

The ionic and water flux rates are asymmetrical along the surfaces and pathway since the total ionic conductance and water permeability are proportional to the surface area (given in Table 1). For the ionic species on each surface or pathway, we will express total ionic conductance as ${g}_{\text {ion},(\cdot )}= \overline{g}_{\text {ion},(\cdot )}\cdot a_{(\cdot )}$, where $\overline{g}_{\text {ion},(\cdot )}$ is the ionic conductance per unit area. In this paper, equations and expressions will use total conductance, while figures will use conductance per unit area.

Active transport mechanisms, specifically the terms ${{\textbf {p}}}_{{{\textbf {ion,eA}}}}$ and ${{\textbf {p}}}_{{{\textbf {ion,AB}}}}$ as seen in Equations (5) and (6), are fixed at 0 in Section 3 while each will be varied in Sections 4-6. Table 4 lists the default values for total NKA pump rates and the stoichiometry coefficients for sodium and potassium on NKA pumps for the basolateral membrane and the apical surface. In this paper, we denote the NKA pump rate per unit area as p. The stoichiometry of the NKA pump is typically found to pump out three Na^+^ for every two K^+^ pumped into the cell. Under some circumstances in nature, the sodium to potassium ratios can differ (Artigas et al. 2023), but, for most animal cells, the values given in Table 4 reduce the electrochemical work performed by the NKA pump (Peluffo and Hernández 2023).

## Passive Two-Compartment Systems

In this section, we present conditions that guarantee the existence of an *equilibrium* in a 10-dimensional *passive* ABp system where there is no active transport.

### Assumption 1

To establish the existence of equilibrium states in passive 2-compartm-ent systems, we impose the following assumptions.

1. No active transport mechanisms are involved, i.e., ${{\textbf {p}}}_{{{\textbf {Na,eA}}}}= {{\textbf {p}}}_{{{\textbf {K,eA}}}}= {{\textbf {p}}}_{{{\textbf {Na,AB}}}}= {{\textbf {p}}}_{{{\textbf {K,AB}}}}= 0$.

2. For each ion, at least two of $g_{\text {ion},eA}, g_{\text {ion},AB}, g_{\text {ion},eB}$ are strictly positive.

3. At least two of $\nu _{eA},\nu _{AB}, \nu _{eB}$ are strictly positive.

4. $$[K]_e,[Na]_e,[Cl]_e,[Y]_e> 0$$.

Note that to achieve a positive and finite volume at equilibrium, the concentration of impermeant molecules, $[X]_j^{eq}$, must be strictly positive. The next lemma provides the conditions for this.

### Lemma 1

For $$j\in \{A,B\}$$, let

9 $$\begin{aligned} {\xi _j} := {\left\{ \begin{array}{ll} \dfrac{\mathcal {O}_e- \sqrt{4\left( 1 - z_j^2 \right) \mathcal {C}_{eq}^2+ \mathcal {O}_e^2 z_j^2}}{1 - z_j^2} & \text { if } z_j^2 \ne 1\\ \dfrac{\mathcal {O}_e^2 - 4\mathcal {C}_{eq}^2}{2 \mathcal {O}_e} & \text { if } z_j^2 = 1. \end{array}\right. } \end{aligned}$$

where

10 $$\begin{aligned} {\mathcal {C}_{eq}^2} = [Cl]_e\left( [Na]_e+ [K]_e\right) {=[Cl]_e\left( [Cl]_e-z_{Y}[Y]_e\right) } . \end{aligned}$$

and assume that $[Y]_e> 0$. Then, $4 \mathcal {C}_{eq}^2- \mathcal {O}_e^2 < 0$ and $\xi _j>0$.

### Proof

Substituting (8a) into the third term of Equation (10) gives

$$\begin{aligned} \mathcal {C}_{eq}^2&= \left( \frac{1}{2}\mathcal {O}_e+ \frac{1}{2}\left( z_{Y}- 1\right) [Y]_e\right) \left( \left( \frac{1}{2}\mathcal {O}_e+ \frac{1}{2}\left( z_{Y}- 1\right) [Y]_e\right) -z_{Y}[Y]_e\right) \\&= \frac{1}{4}\left( \left( \mathcal {O}_e- [Y]_e\right) ^2 - z_{Y}^2[Y]_e^2 \right) . \end{aligned}$$

Hence $4 \mathcal {C}_{eq}^2- \mathcal {O}_e^2=[Y]_e\left( [Y]_e- z_{Y}^2 [Y]_e- 2\mathcal {O}_e\right) .$ Since $0<[Y]_e<\mathcal {O}_e$, we conclude that $4 \mathcal {C}_{eq}^2- \mathcal {O}_e^2<0$. For $z_j^2 = 1$, it is straightforward to show that if $4 \mathcal {C}_{eq}^2- \mathcal {O}_e^2<0$ then $\xi _j >0$. For $z_j^2 < 1$ (>1), since $4 \mathcal {C}_{eq}^2- \mathcal {O}_e^2<0$, we can conclude that $4(1-z_{j}^2)\mathcal {C}_{eq}^2+z_{j}^2\mathcal {O}_e^2 < \mathcal {O}_e^2$, ($> \mathcal {O}_e^2$). Hence $\xi _j >0$. □

### Proposition 1

Consider the coupled PLEs (2)–(6) for 2-compartment systems under Assumption 1. Then, the system admits a stable equilibrium point, given explicitly as follows:

11a $$\begin{aligned}&[Na]_j^{eq}= \dfrac{2 [Na]_e[Cl]_e}{\mathcal {O}_e+ \left( z_j - 1 \right) [X]_j^{eq}}, \end{aligned}$$

11b $$\begin{aligned}&[K]_j^{eq}= \dfrac{2 [K]_e[Cl]_e}{\mathcal {O}_e+ \left( z_j - 1 \right) [X]_j^{eq}}, \end{aligned}$$

11c $$\begin{aligned}&[Cl]_j^{eq}= \frac{1}{2}\left( \mathcal {O}_e+ \left( z_j - 1 \right) [X]_j^{eq}\right) , \end{aligned}$$

11d $$\begin{aligned}&w_j^{eq}= \frac{X_j}{[X]_j^{eq}}, \end{aligned}$$

11e $$\begin{aligned}&V_j^{eq}= \frac{RT}{F}\ln \left( \frac{\mathcal {O}_e+ \left( z_j - 1 \right) [X]_j^{eq}}{2 [Cl]_e} \right) . \end{aligned}$$

where $[X]_j^{eq}= \xi _j$ is given in Equation (9).

### Proof

To obtain the equilibria, we consider Assumption 1.1 and set the right-hand side of Equations (5)–(6) equal to zero:

12a $$\begin{aligned} 0&=-g_{\text {ion},eA}\left( V_A^{eq}-E_{\text {ion},A}^{eq}\right) -g_{\text {ion},AB}\left( \left( V_A^{eq}-E_{\text {ion},A}^{eq}\right) -\left( V_B^{eq}-E_{\text {ion},B}^{eq}\right) \right) \end{aligned}$$

12b $$\begin{aligned} 0&=-g_{\text {ion},eB}\left( V_B^{eq}-E_{\text {ion},B}^{eq}\right) +g_{\text {ion},AB}\left( \left( V_A^{eq}-E_{\text {ion},A}^{eq}\right) -\left( V_B^{eq}-E_{\text {ion},B}^{eq}\right) \right) . \end{aligned}$$

By summing (12a) and (12b), and assuming that at least one of $g_{\text {ion},eA}$ or $g_{\text {ion},AB}$ is strictly positive (Assumption 1.2), we can express $V_A^{eq} - E_{\text {ion},A}^{eq}$ in terms of $V_B^{eq} - E_{\text {ion},B}^{eq}$, or vice versa. We can then use (12a) or (12b) to solve for both quantities. For example, assume that $g_{\text {ion},eA} > 0$, then

13 $$\begin{aligned} V_A^{eq}-E_{\text {ion},A}^{eq}=-\frac{g_{\text {ion},AB}}{g_{\text {ion},eA}}\left( V_B^{eq}-E_{\text {ion},B}^{eq}\right) . \end{aligned}$$

Substituting (13) into (12a) yields

$$\begin{aligned} 0=\left( g_{\text {ion},eA}g_{\text {ion},AB}+g_{\text {ion},{eA}}g_{\text {ion},eB}+g_{\text {ion},AB}g_{\text {ion},eB}\right) \left( V_B^{eq}-E_{\text {ion},B}^{eq}\right) . \end{aligned}$$

Since by Assumption 1.2, the first term of the product is non-zero, we get $V_B^{eq}-E_{\text {ion},B}^{eq}=0,$ and hence, we conclude that for $j\in \{A,B\}$, the following electrochemical potential condition holds for sodium, potassium, and chloride:

14 $$\begin{aligned}&V_j^{eq}= E_{\text {ion},j}^{eq}. \end{aligned}$$

At equilibrium, the chemical and electrical potentials counterbalance each other, leaving the system without a net flux of ions.

Next, setting the right-hand side of Equation (2) to zero we obtain

15a $$\begin{aligned}&0=\nu _{eA}\left( \mathcal {O}_A^{eq}-\mathcal {O}_e\right) +\nu _{AB}\left( \mathcal {O}_A^{eq}-\mathcal {O}_B^{eq}\right) \end{aligned}$$

15b $$\begin{aligned}&0=\nu _{eB}\left( \mathcal {O}_B^{eq}-\mathcal {O}_e\right) -\nu _{AB}\left( \mathcal {O}_A^{eq}-\mathcal {O}_B^{eq}\right) . \end{aligned}$$

By summing (15a) and (15b), and assuming that at least one of $\nu _{eA}$ or $\nu _{eB}$ is strictly positive, we can express $\mathcal {O}_A^{eq}-\mathcal {O}_e$ in terms of $\mathcal {O}_B^{eq}-\mathcal {O}_e$, or vice versa. We can then use (15a) or (15b) to solve for both quantities. For example, assume that $\nu _{eA}>0$. Then,

16 $$\begin{aligned} \mathcal {O}_A^{eq}-\mathcal {O}_e=-\frac{\nu _{eB}}{\nu _{eA}}\left( \mathcal {O}_B^{eq}-\mathcal {O}_e\right) . \end{aligned}$$

Substituting (16) into (15a) yields $\left( \nu _{eA}\nu _{AB}+\nu _{eA}\nu _{eB}+\nu _{AB}\nu _{eB}\right) \left( \mathcal {O}_B^{eq}-\mathcal {O}_e\right) =0,$ and hence, for $j\in \{A,B\}$, we get $\mathcal {O}_j^{eq}= \mathcal {O}_e$ or equivalently,

17 $$\begin{aligned} [Na]_j^{eq}+[K]_j^{eq}+[Cl]_j^{eq}+[X]_j^{eq}= \mathcal {O}_e. \end{aligned}$$

At equilibrium, the compartments have equal osmolarity. As a result, the surfaces and pathways lack any net water flow. Although the osmolarities are the same in both spaces, the solute composition of each compartment may differ (Boron and Boulpaep 2016).

Electroneutrality requires

18 $$\begin{aligned}&[Na]_j^{eq}+[K]_j^{eq}-[Cl]_j^{eq}+z_j[X]_j^{eq}= 0 . \end{aligned}$$

Adding Equations (17) and (18) gives us

19 $$\begin{aligned}&[Na]_j^{eq}+ [K]_j^{eq}= \frac{1}{2}\left( \mathcal {O}_e- \left( z_{j} + 1 \right) [X]_j^{eq}\right) . \end{aligned}$$

Subtracting Equation (18) from Equation (17) gives us the equilibrium value of chloride as in (11c).

From Equation (14), we can express the intracellular electrical potential in terms of $[Cl]_j^{eq}$, which gives us $V_j^{eq}= -\frac{RT}{F}\ln \left( \frac{[Cl]_e}{[Cl]_j^{eq}}\right)$. Chloride concentration $[Cl]_j^{eq}$ can be substituted with the right-hand side of Equation (11c). This allows us to describe the equilibrium value of electrical potential in terms of $[X]_j^{eq}$, as given in (11e).

The intracellular sodium and potassium concentrations $[Na]_j$ and $[K]_j$ are isolated from the potential in Equation (14), and expressed as

20 $$\begin{aligned} [\text {ion}]_j^{eq}&= [\text {ion}]_e \exp \left( -\frac{z_{\text {ion}}F}{RT} V_j^{eq}\right) . \end{aligned}$$

Notice that both $[Na]_j^{eq}$ and $[K]_j^{eq}$ depend on the $V_j^{eq}$, which in turn depends on $[X]_j^{eq}$ as expressed in (11a)–(11b). Finally, by definition, the equilibrium value of the volume can be expressed as (11d).

To finish the proof of existence, we now solve for the impermeant concentration $[X]_j^{eq}$. The ionic concentrations on the left-hand side of Equation (19) can be replaced with the expressions found on the right-hand side of Equation (20). The voltage in Equation (19) can be replaced with the right-hand side of Equation (11e). This gives us

21 $$\begin{aligned} \frac{1}{2}\left( \mathcal {O}_e- \left( 1+z_j \right) [X]_j^{eq}\right)&= [Na]_e\exp \left( -\ln \left( \frac{\mathcal {O}_e+ \left( z_j - 1 \right) [X]_j^{eq}}{2 [Cl]_e} \right) \right) \nonumber \\&\quad + [K]_e\exp \left( -\ln \left( \frac{\mathcal {O}_e+ \left( z_j - 1 \right) [X]_j^{eq}}{2 [Cl]_e} \right) \right) . \end{aligned}$$

Solving for $[X]_j^{eq}$ in Equation (21) gives us an expression for impermeant concentration as given in (9). Note that Assumption 1.4 and Lemma 1 guarantee that $[X]_j^{eq}$ is strictly positive and hence the corresponding volume is finite. The rest of Equation (11) can be derived from (20) and the fact that $w_j=X_j/[X_j]$.

The proof of stability is identical to that of the active 2-compartment system with p=0. We will discuss this case in detail in Section 4.2.

□

Equation (11) provides analytical expressions for the equilibrium solutions of the coupled PLEs described in Section 2. The expressions for each compartment depend on the shared extracellular concentrations as well as on the number of impermeant molecules, $X_j$, and their average charges, $z_{j}$.

If these compartment-specific parameters are identical, i.e., $X_A= X_B$ and $z_{A}= z_{B}$, then the equilibrium states of the two compartments become indistinguishable, and the ABp system behaves like a single compartment at equilibrium. See Aminzare and Kay (2024) for more details on the single-cell case.

Note that the equilibrium volume $w_j^{eq}$ is the only state variable that depends on the number of impermeant molecules $X_j$, and the equilibrium values for the ionic concentrations and voltages only depend on $z_{j}$. A well-defined, locally asymptotically stable equilibrium requires impermeant molecules in the external compartment, i.e., $[Y]_e>0$, to provide the osmotic constraint needed for volume regulation. As mentioned before, for $z_{A}= z_{B}$, the equilibrium values of ion concentrations and voltages of compartments *A* and *B* are identical, and their volumes depend on $X_A$ and $X_B$.

## Active Two-Compartment Systems: NKA Pump on Basolateral Membrane

In this section, we add Na^+^/K^+^-ATPase (NKA) pumps to membranes and derive the steady states of coupled PLEs analytically. In most epithelial systems, the NKA pump is confined to the basolateral membrane or on the lateral borders of epithelial cells (Shoshani et al. 2005). In rarer cases, the NKA pump is expressed on the apical surface (Pollay et al. 1985). We first consider the NKA pump on the basolateral membrane and later in Section 6, we will explore the effect of the NKA pump on the apical surface. An active NKA pump mechanism will pump $\gamma _{Na}$ Na^+^ from the intracellular space to the extracellular space for every $\gamma _{K}$ K^+^ ions pumped into the intracellular space. Here, $\gamma _{Na}$ and $\gamma _{K}$ denote the stoichiometries of the NKA pump – typically 3 and 2, respectively (Peluffo and Hernández 2023). Active transport by the basolateral NKA directly regulates ionic concentrations in compartment *A* and, through transcellular coupling and paracellular leak, also influences concentrations in compartment *B*. We will use Equations (5)–(6) with the *constant* active pump mechanism given by ${{\textbf {p}}}_{{{\textbf {Na,eA}}}}= -\gamma _{Na} \, p\, a_{eA}, {{\textbf {p}}}_{{{\textbf {K,eA}}}}= \gamma _{K} \, p\, a_{eA}, {{\textbf {p}}}_{{{\textbf {Na,AB}}}}={{\textbf {p}}}_{{{\textbf {K,AB}}}}= 0,$ where p and $a_{eA}$ are non-negative constants. The schematic diagrams for ABp systems in Figure 1(right) illustrate this configuration, with the NKA pump located on the interface between the extracellular space and compartment *A*. Passive transport mechanisms are the sole means of ion transport at the confluence of compartments *A* and *B*, effectively coupling the two compartments and enabling the NKA pump to influence both. The ion composition observed in compartment *B* almost mirrors that in compartment *A*, while volume changes are also proportional.

Note that the constant NKA pump used here represents the simplest form. More complex nonlinear formulations exist, such as

22 $$\begin{aligned} p\, a_{eA}\left( \frac{[K]_e}{k_p+[K]_e}\right) ^2\left( \frac{[Na]_A}{k_{Na}+[Na]_A}\right) ^3, \end{aligned}$$

first introduced in Garay and Garrahan (1973), where $k_p = 0.883$ mM and $k_{Na} = 3.56$ mM are the apparent dissociation constants for $\hbox {K}^+$ and $\hbox {Na}^+$, respectively. Simpler nonlinear forms have also been proposed, including $p\, a_{eA}[Na]_A$ (Yurinskaya et al. 2020), $p\, a_{eA}\left( \tfrac{[Na]_A}{[Na]_e}\right) ^3$ (Keener and Sneyd 2009), and $p\, a_{eA}\left( \tfrac{[K]_e}{[K]_A}\right) ^2\left( \tfrac{[Na]_A}{[Na]_e}\right) ^3$ (Manicka and Levin 2019). In Aminzare and Kay (2024), Aminzare and Kay analytically demonstrated that the exact mathematical form of the NKA pump does not qualitatively alter the steady states of a single cell; only the stoichiometry of the pump affects them.

**Fig. 2:**
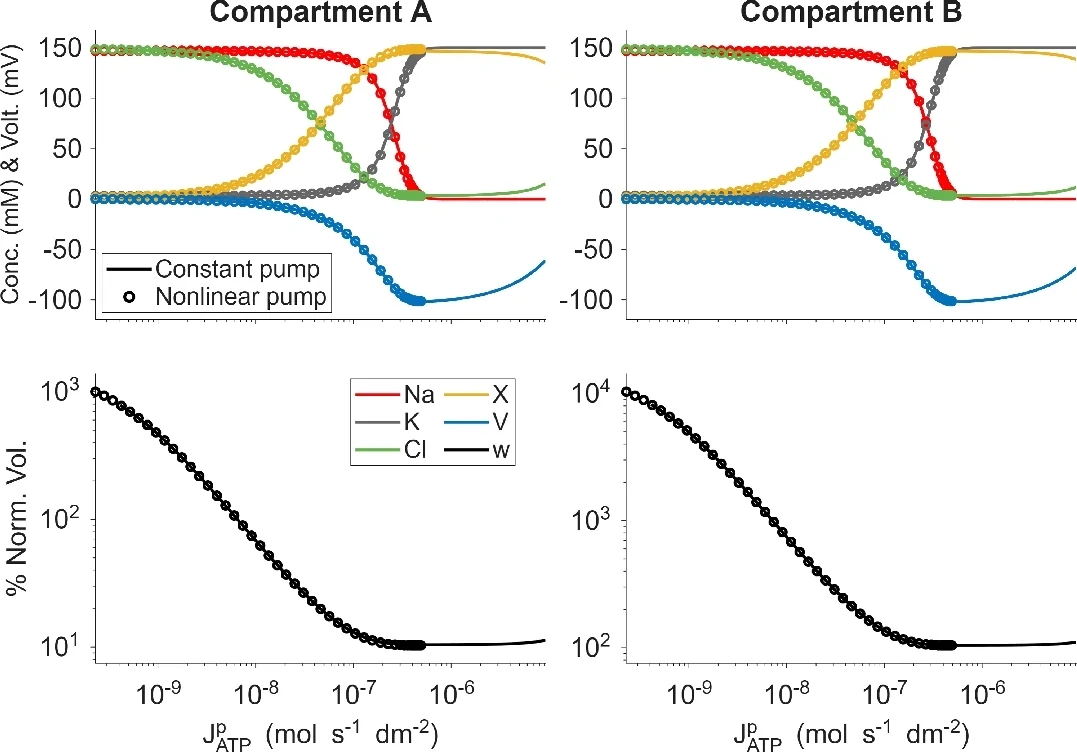
Steady-state values of the ABp system are plotted against ATP consumption rate for the constant NKA (solid curve) and the nonlinear NKA (22) (circles) (color figure online)

The hydrolysis of ATP (Adenosine Triphosphate) releases free energy that drives a wide range of cellular processes, including active ion transport (Dunn and Grider 2025). The rate at which the NKA pump operates determines the ATP consumption rate. In particular, the ATP consumption rate for a constant NKA pump is defined as $J_{ATP}^{p}={p\, a_{eA}}/{F}$. For the nonlinear NKA pump in Equation (22), this rate is $J_{ATP}^{p}= \text {(22)}/F$. In Figure 2, we plot the steady states of the ABp system as a function of the ATP consumption rate for both the constant and nonlinear models of the NKA pump. As the figure shows, the steady state values are qualitatively similar, consistent with those found in single cells (Aminzare and Kay 2024). Therefore, it is reasonable to use a constant pump, as it does not alter the qualitative behavior of the steady states and is more mathematically tractable. While we use analytical expressions for the steady states in the constant pump case (derived in Section 4.1 below), we compute them numerically for the nonlinear pump models.

### Existence of the steady states of coupled PLEs

To establish the existence of steady states (denoted by the superscript ^*ss*^) in 2-compartment systems where the NKA pump is located on basolateral membrane, we prove the following lemma which will provide a possible range for the pump rate p and impose the following assumptions.

#### Lemma 2

For ions Na^+^ and K^+^, assume that at least two of $g_{\text {ion},{eA}}$, $g_{\text {ion},{AB}}$, and $g_{\text {ion},{eB}}$ are strictly positive. For $j\in \{A,B\}$ let

23 $$\begin{aligned} {\mathcal {C}_{j}^2}&= [Cl]_e\left( [Na]_ee^{- \frac{F}{RT}\gamma _{Na} \, p\, a_{eA}G_{Na,j}} + [K]_ee^{\frac{F}{RT} \gamma _{K} \, p\, a_{eA}G_{K,j}} \right) , \end{aligned}$$

where the constants $G_{\text {ion},j}$ are defined as

24a $$\begin{aligned}&G_{\text {ion},A}= \dfrac{g_{\text {ion},AB}+ g_{\text {ion},eB}}{g_{\text {ion},eA}\, g_{\text {ion},AB}+ g_{\text {ion},eA}\, g_{\text {ion},eB}+ g_{\text {ion},AB}\, g_{\text {ion},eB}} \end{aligned}$$

24b $$\begin{aligned}&G_{\text {ion},B}= \dfrac{g_{\text {ion},AB}}{g_{\text {ion},eA}\, g_{\text {ion},AB}+ g_{\text {ion},eA}\, g_{\text {ion},eB}+ g_{\text {ion},AB}\, g_{\text {ion},eB}} . \end{aligned}$$

Then, if

25 $$\begin{aligned} \gamma _{Na}[Na]_eG_{Na,j}- \gamma _{K}[K]_eG_{K,j}> 0 , \end{aligned}$$

there exists $p_{\max }>0$ such that for $p<p_{\max }$,

26 $$\begin{aligned} 4\mathcal {C}_{j}^2-\mathcal {O}_e^2<0. \end{aligned}$$

Hence, for $0<p<p_{\max }$, $[X]_j^{ss}>0$, where

27 $$\begin{aligned} [X]_j^{ss}&= {\left\{ \begin{array}{ll} \dfrac{\mathcal {O}_e- \sqrt{4\left( 1 - z_{j}^2 \right) \mathcal {C}_{j}^2+ \mathcal {O}_e^2 z_{j}^2}}{1 - z_{j}^2} & \text { if } z_{j}^2 \ne 1\\ \dfrac{\mathcal {O}_e^2 - 4\mathcal {C}_{j}^2}{2 \mathcal {O}_e} & \text { if } z_{j}^2 = 1 . \end{array}\right. } \end{aligned}$$

In Lemma 2, the conditions on $g_{\text {ion},{eA}}$, $g_{\text {ion},{AB}}$, and $g_{\text {ion},{eB}}$ ensure that the denominator of $G_{\text {ion},j}$ is non-zero. For instance, it is permissible to have $g_{Na,eA}= g_{K,AB}= 0$, provided that $g_{Na,AB}\;g_{Na,eB}> 0$ and $g_{K,eA}\; g_{K,eB}> 0$.

Note that it is possible to assume $\overline{g}_{\text {ion},AB}= 0$, in which case compartments *A* and *B* become decoupled–*A* transport mechanism remains active while *B* is passive. Although we exclude this case from our analysis, we note that the steady state values for *A* and the equilibrium values for *B* are consistent with those derived in previous studies (Aminzare and Kay 2024).

#### Proof

To show that there exists a $p_{\max }> 0$ such that for all $p< p_{\max }$ we have

$$ f_{j,{eA}} (p) := 4\mathcal {C}_{j}^2- \mathcal {O}_e^2 < 0, $$

we need to verify the following two conditions: (1) $f_{j,{eA}}(0) \le 0$, and (2) $f'_{j,{eA}}(0) < 0$. Then, since $f_{j,{eA}}$ is continuous, we conclude that $f_{j,{eA}}(p) < 0$ for all sufficiently small values of p>0. We denote the supremum of such values by $p_{\max }$.

(1) Note that $f_{j,{eA}}(0) = 4\mathcal {C}_{eq}^2- \mathcal {O}_e^2 = [Y]_e([Y]_e- z_{Y}^2 [Y]_e- 2\mathcal {O}_e),$ which, by Lemma 1, is negative for $[Y]_e> 0$ and is zero when $[Y]_e= 0$.

(2) Next, we compute the derivative:

$$\begin{aligned} f'_{j,{eA}}(p) = \frac{F4[Cl]_ea_{eA}}{RT}&\left( -[Na]_e\gamma _{Na} G_{Na,j}e^{\frac{F}{RT} \gamma _{Na} pa_{eA}G_{Na,j}} \right. \\&\left. + [K]_e\gamma _K G_{K,j}e^{\frac{F}{RT} \gamma _K pa_{eA}G_{K,j}} \right) . \end{aligned}$$

Evaluating at p=0, we obtain $f'_{j,{eA}}(0) = 4[Cl]_e\frac{F}{RT} a_{eA}\left( -[Na]_e\gamma _{Na} G_{Na,j}\right. \left. + [K]_e\gamma _K G_{K,j}\right) .$ By (25), the term in parentheses is negative, and hence $f'_{j,{eA}}(0) < 0$.

A similar argument to that in Lemma 1 shows that $[X]_j^{ss} > 0$.

□

#### Proposition 2

Consider the coupled PLEs (2)–(6) for 2-compartment systems with

$$\begin{aligned} {{\textbf {p}}}_{{{\textbf {Na,eA}}}}= -\gamma _{Na} \, p\, a_{eA}, {{\textbf {p}}}_{{{\textbf {K,eA}}}}= \gamma _{K} \, p\, a_{eA}, {{\textbf {p}}}_{{{\textbf {Na,AB}}}}={{\textbf {p}}}_{{{\textbf {K,AB}}}}= 0. \end{aligned}$$

Under the assumptions of Lemma 2, and the following two assumptions:

1. at least two of $g_{\text {Cl},{eA}}$, $g_{\text {Cl},{AB}}, g_{\text {Cl},{eB}}$ are strictly positive
2. at least two of $\nu _{eA},\nu _{AB},\nu _{eB}$ are strictly positive

the system admits a stable steady state for $0<p<p_{\max }$ , where $p_{\max }$ is defined in Lemma 2. The steady states can be described explicitly as follows. For $j\in \{A,B\}$:

28a $$\begin{aligned}&[Na]_j^{ss} = \dfrac{2 [Na]_e[Cl]_e\exp \left( - \frac{F}{RT}\gamma _{Na} \, p\, a_{eA}G_{Na,j}\right) }{\mathcal {O}_e+ \left( z_j - 1 \right) [X]_j^{ss}} \end{aligned}$$

28b $$\begin{aligned}&[K]_j^{ss} = \dfrac{2 [K]_e[Cl]_e\exp \left( \frac{F}{RT} \gamma _{K} \, p\, a_{eA}G_{K,j}\right) }{\mathcal {O}_e+ \left( z_j - 1 \right) [X]_j^{ss}} \end{aligned}$$

28c $$\begin{aligned}&[Cl]_j^{ss} = \frac{1}{2}\left( \mathcal {O}_e+ \left( z_j - 1 \right) [X]_j^{ss} \right) \end{aligned}$$

28d $$\begin{aligned}&w_j^{ss} = \frac{X_j}{[X]_j^{ss}} \end{aligned}$$

28e $$\begin{aligned}&V_j^{ss} = \frac{RT}{F}\ln \left( \frac{\mathcal {O}_e+ \left( z_j - 1 \right) [X]_j^{ss}}{2 [Cl]_e} \right) , \end{aligned}$$

where $G_{\text {ion},A},G_{\text {ion},B}$ are given in (24) and $[X]_j^{ss}$ is given in (27).

Note that, unlike in the passive case, $[Y]_e$ is not required to be positive. Indeed, as long as the NKA pump remains active, it compensates for $[Y]_e$ and maintains regulation of the 2-compartment system.

#### Proof

To derive the steady states, we set the right-hand side of Equations (5) and (6) to zero. For sodium, this yields:

29a $$\begin{aligned}&0=-g_{Na,eA}\left( V_A^{ss}-E_{Na,A}^{ss}\right) - g_{Na,AB}\left( \left( V_A^{ss}-E_{Na,A}^{ss}\right) -\left( V_B^{ss}-E_{Na,B}^{ss}\right) \right) -\gamma _{Na} \, p\, a_{eA} \end{aligned}$$

29b $$\begin{aligned}&0=-g_{Na,eB}\left( V_B^{ss}-E_{Na,B}^{ss}\right) +g_{Na,AB}\left( \left( V_A^{ss}-E_{Na,A}^{ss}\right) -\left( V_B^{ss}-E_{Na,B}^{ss}\right) \right) . \end{aligned}$$

Summing (29a) and (29b), and assuming that at least one of $g_{Na,eA}$, $g_{Na,eB}$ is non-zero, we can solve for the electrochemical potential difference in the corresponding compartment. For instance if $g_{Na,eA}>0$, then:

$$\begin{aligned} V_A^{ss} - E_{Na,A}^{ss} = \frac{-g_{Na,eB}\left( V_B^{ss} - E_{Na,B}^{ss}\right) - \gamma _{Na} \, p\, a_{eA}}{g_{Na,eA}}. \end{aligned}$$

Substituting this expression into Equation (29b) yields

30 $$\begin{aligned} 0=&-(V_B^{ss}-E_{Na,B}^{ss})(g_{Na,eA}g_{Na,AB}+g_{Na,eA}g_{Na,eB}+g_{Na,AB}g_{Na,eB}) \nonumber \\&- g_{Na,AB}\gamma _{Na} \, p\, a_{eA}. \end{aligned}$$

We can now solve for $V_B^{ss}-E_{Na,B}^{ss}$ and then for $V_A^{ss}-E_{Na,A}^{ss}$. Similarly, we can solve for the electrochemical potential of potassium and chloride in each compartment. In summary, for $j\in \{A,B\}$, the electrochemical potential of sodium, potassium, and chloride become:

31a $$\begin{aligned}&V_j^{ss} - E_{Na,j}^{ss} = -\gamma _{Na} \, p\, a_{eA}G_{Na,j}=: \eta _{Na,j}\end{aligned}$$

31b $$\begin{aligned}&V_j^{ss} - E_{K,j}^{ss} = \gamma _{Na} \, p\, a_{eA}G_{K,j}=: \eta _{K,j}\end{aligned}$$

31c $$\begin{aligned}&V_j^{ss} - E_{Cl,j}^{ss} = 0 =: \eta _{Cl,j} \end{aligned}$$

where, for ions Na^+^ and K^+^, the constants $G_{\text {ion},j}$ are defined in (24) as

$$\begin{aligned}&G_{\text {ion},A}= \dfrac{g_{\text {ion},AB}+ g_{\text {ion},eB}}{g_{\text {ion},eA}\, g_{\text {ion},AB}+ g_{\text {ion},eA}\, g_{\text {ion},eB}+ g_{\text {ion},AB}\, g_{\text {ion},eB}} \\&G_{\text {ion},B}= \dfrac{g_{\text {ion},AB}}{g_{\text {ion},eA}\, g_{\text {ion},AB}+ g_{\text {ion},eA}\, g_{\text {ion},eB}+ g_{\text {ion},AB}\, g_{\text {ion},eB}} . \end{aligned}$$

We denote the right-hand side of the expressions in Equation (31) by $\eta _{\text {ion},j}$. By isolating the Nernst potentials in Equation (31) and solving for the intracellular concentrations $[\text {ion}]_j^{ss}$, we obtain the following expression for $[\text {ion}]_j^{ss}$ in terms of $V_j^{ss}$:

32 $$\begin{aligned} [\text {ion}]_j^{ss} = [\text {ion}]_e \exp \left( \frac{F}{RT}\left( \eta _{\text {ion},j}- V_j^{ss} \right) \right) . \end{aligned}$$

Next, we derive an expression for $V_j^{ss}$ in terms of fixed parameters. Since the addition of the pump mechanism does not affect the volume equations, we can proceed similarly to the previous section and derive Equation (19) for the steady state values using the expression for $[\text {ion}]_j^{ss}$ given in Equation (32):

33 $$\begin{aligned} [Na]_j^{ss} + [K]_j^{ss} = \frac{1}{2}\left( \mathcal {O}_e- \left( z_{j} + 1 \right) [X]_j^{ss} \right) . \end{aligned}$$

Similarly, since $V_j^{ss} = E_{\text {Cl},j}^{ss}$, the voltage $V_j^{ss}$ can be derived analogously to Equation (11e), resulting in Equation (28e).

Substituting Equations (28e) and (32) into Equation (33) yields:

$$\begin{aligned} \begin{aligned} \frac{\mathcal {O}_e- \left( 1 + z_j \right) [X]_j^{ss}}{2} = \;&[Na]_e\exp \left( \frac{F}{RT} \left( \eta _{Na,j}- \frac{RT}{F} \ln \left( \frac{\mathcal {O}_e+ \left( z_j - 1 \right) [X]_j^{ss}}{2 [Cl]_e} \right) \right) \right) \\&+[K]_e\exp \left( \frac{F}{RT} \left( \eta _{K,j}- \frac{RT}{F} \ln \left( \frac{\mathcal {O}_e+ \left( z_j - 1 \right) [X]_j^{ss}}{2 [Cl]_e} \right) \right) \right) , \end{aligned} \end{aligned}$$

which simplifies to Equation (27) with the definition in Equation (23). Since $0< p< p_{\max }$, Lemma 2 guarantees that $[X]_j^{ss}$ is strictly positive, ensuring the steady states are well-defined.

The remainder of Equation (28) follows from Equations (28e), (32), and the identity $w_j = X_j/ [X_j]$.

The proof of stability is discussed in detail in Section 4.2.

□

Cellular volume can be challenging to compare across figures because it is measured in liters and varies widely in magnitude between systems. To address this, we normalize volume by $w_{\text {Norm}}$, defined as $w_A^{ss}$ evaluated at default parameter values with the pump rate fixed at p=1 μA $\hbox {dm}^{-2}$ on the basolateral membrane:34

#### A note on the possible range of the pump rate

In Lemma 2, we showed that if $\gamma _{Na}[Na]_eG_{Na,j}- \gamma _{K}[K]_eG_{K,j}> 0$, then there exists a range for the pump rate per unit area p, denoted by $(0,p_{\max })$, for which steady states exist and, in particular, the compartment volumes remain finite. Define

35 $$\begin{aligned} f_{j,{eA}}\left( p\right) := 4 \, \mathcal {C}_{j}^2(p)-\mathcal {O}_e^2. \end{aligned}$$

**Fig. 3:**
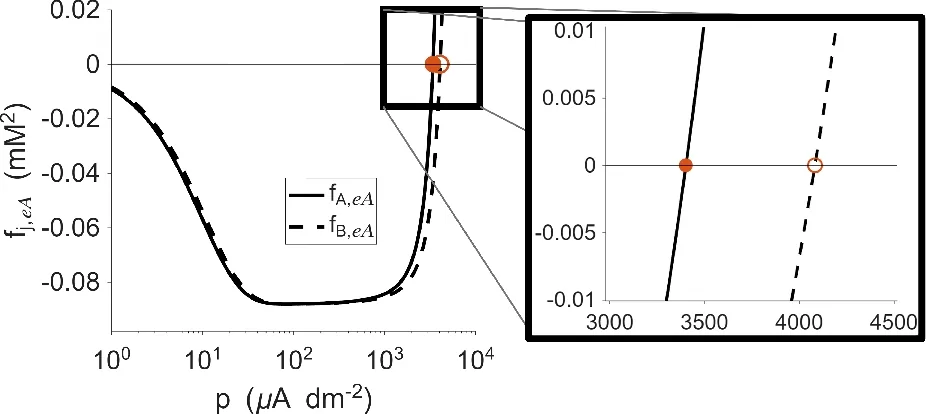
The admissible range of p is computed numerically by plotting $f_{j,{eA}}(p):= 4 \, \mathcal {C}_{j}^2(p) - \mathcal {O}_e^2$ and identifying the interval $(0, p_{\max })$ where $f_{j,{eA}}(p) < 0$. In the zoomed-in panel, the filled and open circles denote the roots of $f_{A,{eA}}$ and $f_{B,{eA}}$, respectively (color figure online)

Our default parameter values are chosen so that (25) holds. Therefore, we expect that $f_{j,{eA}}(p) < 0$ over some interval of p. Figure 3 shows the plots of $f_{j,{eA}}(p)$ for compartments *A* and *B*, which appear nearly identical. The right panel provides a zoomed-in view near their positive roots, denoted $p_{\max ,A}$ and $p_{\max ,B}$. Solving for these roots numerically yields $p_{\max ,A} \approx 3,402$ μA $\hbox {dm}^{-2}$ and $p_{\max ,B} \approx 4,082$ μA $\hbox {dm}^{-2}$. In the simulations presented in the following sections, we fix $p_{\max }= \min \{p_{\max ,A}, p_{\max ,B}\}$ and vary p within the range $(0, p_{\max })$.

The pump rate p, denoted by $p_{\min }$, is defined as the value of p that minimizes the steady-state compartment volume $w_j^{ss}$. To compute $p_{\min }$, we differentiate $w_j^{ss}(p)$ from (28d) with respect to p and solve for the value at which the derivative is zero. For any $z_j$ and $j \in \{A, B\}$, the minimizing pump rate $p_{\min ,j}$ is given by:

36 $$\begin{aligned} p_{\min ,j}&:= \frac{RT}{Fa_{eA}} \left( \dfrac{1}{\gamma _K G_{K,j}+ \gamma _{Na} G_{Na,j}} \right) \ln \left( \dfrac{\gamma _{Na} [Na]_eG_{Na,j}}{\gamma _{K} [K]_eG_{K,j}} \right) . \end{aligned}$$

For $p_{\min ,j}$ to be positive, the argument of the logarithm must exceed 1; that is, $\frac{\gamma _{Na} [Na]_eG_{Na,j}}{\gamma _{K} [K]_eG_{K,j}}>1,$ which is precisely the condition in Equation (25), a necessary condition for the existence of steady states. An analogous condition has also been used for single-compartment models in previous studies (Mori 2012; Aminzare and Kay 2024). Finally, we note that $p_{\min ,j}$ depends on the sodium and potassium conductances, the stoichiometry of the NKA pump, and the extracellular sodium and potassium concentrations. Since the values of $p_{\min ,j}$ play a key role in understanding the system’s behavior, we compute them numerically using our default parameter values and use them in the simulations presented in the following sections: $p_{\min ,A} \approx 80 \mu \text {A dm}^{-2} \text {and} p_{\min ,B} \approx 90 \mu \text {A dm}^{-2}.$

#### A note on ion flows of a ABp system at steady states

For an ABp system with the NKA pump located on the basolateral surface, the ionic fluxes across the three interfaces are

37a $$\begin{aligned}&\Phi _{\text {ion},eA}= z_{\text {ion}}g_{\text {ion},eA}\left( V_A- E_{\text {ion},A}\right) + {{\textbf {p}}}_{{{\textbf {ion,eA}}}}, \end{aligned}$$

37b $$\begin{aligned}&\Phi _{\text {ion},AB}= - z_{\text {ion}}g_{\text {ion},AB}\left[ \left( V_A- E_{\text {ion},A}\right) - \left( V_B- E_{\text {ion},B}\right) \right] , \end{aligned}$$

37c $$\begin{aligned}&\Phi _{\text {ion},eB}= - z_{\text {ion}}g_{\text {ion},eB}\left( V_B- E_{\text {ion},B}\right) , \end{aligned}$$

where $$ {{\textbf {p}}}_{{{\textbf {Na,eA}}}}=-\gamma _{Na} \, p\, a_{eA}$$, $${{\textbf {p}}}_{{{\textbf {K,eA}}}}= \gamma _{K} \, p\, a_{eA},$$ and $${{\textbf {p}}}_{{{\textbf {Cl,eA}}}}=0.$$

**Fig. 4:**
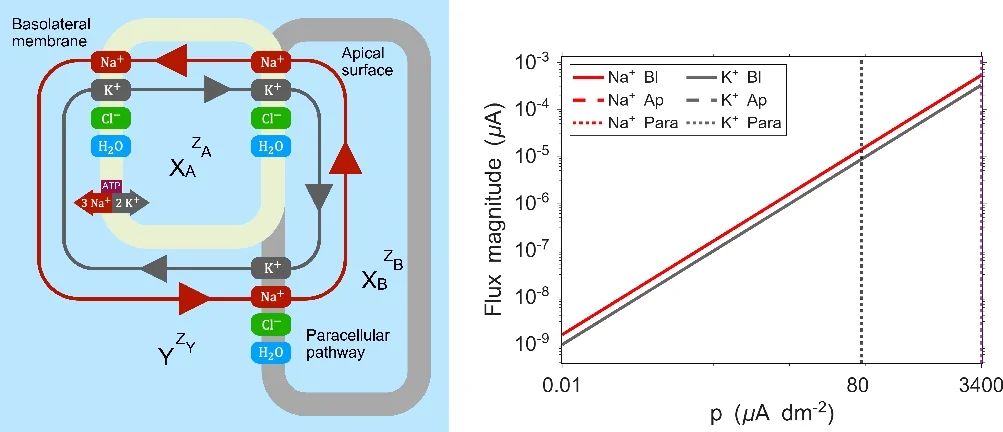
Sodium and potassium flow across the membranes at steady state. The arrows in the left schematic of the ABp system indicate counterclockwise sodium and clockwise potassium transport loops. Sodium moves from *B* to *A* across the apical surface, exits *A* via the basolateral membrane, and returns to *B* through the paracellular pathway, whereas potassium follows the opposite loop. The right panel shows the magnitudes of the total sodium (red) and potassium (gray) fluxes across the three interfaces increase with the pump rate p, with $\hbox {Na}^+$ fluxes slightly exceeding $\hbox {K}^+$ fluxes and remaining equal across their respective interfaces. Here, total flux denotes the area-integrated flux (in μA), not normalized by membrane or pathway area (color figure online)

Using the steady state values derived in Equation (28), we show that the absolute values of total ion and water flows remain equal across the apical, basolateral, and paracellular pathways, that is, $\Phi _{\text {ion},eA}^{ss} = \Phi _{\text {ion},AB}^{ss} = \Phi _{\text {ion},eB}^{ss}.$ As illustrated in Figure 4 (left panel), sodium flow forms a counterclockwise loop: sodium moves from compartment *B* to *A* through the apical surface, exits compartment *A* through the basolateral membrane, and returns to compartment *B* via the paracellular pathway, whereas potassium flow follows the opposite (clockwise) direction. The total fluxes across these three membranes are plotted in the right panel as functions of the pump rate p. As p increases, the total flux magnitude across each interface increases while remaining equal, confirming conservation of flow at the steady states. As with sodium, the absolute value of the total potassium flow increases with the NKA pump rate p, as seen in the right panel. Moreover, due to the 3 : 2 pump stoichiometry, the magnitude of the $\hbox {Na}^+$ flux is slightly larger than that of the $\hbox {K}^+$ flux over the plotted range at each interface. The flows of chloride and water remain zero across all membranes (data not shown).

### Local stability analysis of the steady states of coupled PLEs

In this section, we show that the ABp system’s steady state is locally stable, i.e., the eigenvalues of the corresponding Jacobian matrix of PLEs evaluated at the steady state derived in Proposition 2 have negative real parts.

We first perform a change of variable and rewrite PLEs in these new variables. Let

$$\begin{aligned}&{\mathcal {X}}_A=Fw_A[Na]_A,\; {\mathcal {Y}}_A=Fw_A[K]_A,\; {\mathcal {Z}}_A=Fw_A[Cl]_A,\; {\mathcal {W}}_A=Fw_A, \\&{\mathcal {X}}_B=Fw_B[Na]_B,\; {\mathcal {Y}}_B=Fw_B[K]_B, \; {\mathcal {Z}}_B=Fw_B[Cl]_B, \; {\mathcal {W}}_B=Fw_B. \end{aligned}$$

Here, $\mathcal {X}_i$, $\mathcal {Y}_i$, and $\mathcal {Z}_i$ have units of coulombs and represent the signed amounts of ionic charge per species in each compartment. In the new variables, the PLEs become:

38a $$\begin{aligned}&\frac{d \mathcal {X}_A}{dt} = -g_{Na,eA}\left( V_A- E_{Na,A}\right) + g_{Na,AB}\left( (V_B- E_{Na,B})-(V_A- E_{Na,A})\right) - \gamma _{Na} \, p\, a_{eA}, \end{aligned}$$

38b $$\begin{aligned}&\frac{d \mathcal {Y}_A}{dt} = -g_{K,eA}\left( V_A- E_{K,A}\right) + g_{K,AB}\left( (V_B- E_{K,B})-(V_A- E_{K,A})\right) + \gamma _{K} \, p\, a_{eA}, \end{aligned}$$

38c $$\begin{aligned}&\frac{d \mathcal {Z}_A}{dt} = g_{Cl,eA}\left( V_A- E_{Cl,A}\right) -g_{Cl,AB}\left( (V_B- E_{Cl,B})-(V_A- E_{Cl,A})\right) , \end{aligned}$$

38d $$\begin{aligned}&\frac{ d \mathcal {W}_A}{ dt } = \nu _{eA}(\mathcal {O}_A-\mathcal {O}_e) + \nu _{AB} (\mathcal {O}_A-\mathcal {O}_B) , \end{aligned}$$

38e $$\begin{aligned}&\frac{d \mathcal {X}_B }{dt} = -g_{Na,eB}\left( V_B- E_{Na,B}\right) +g_{Na,AB}\left( (V_A- E_{Na,A})-(V_B- E_{Na,B})\right) , \end{aligned}$$

38f $$\begin{aligned}&\frac{d\mathcal {Y}_B}{dt} = -g_{K,eB}\left( V_B- E_{K,B}\right) +g_{K,AB}\left( (V_A- E_{K,A})-(V_B- E_{K,B})\right) , \end{aligned}$$

38g $$\begin{aligned}&\frac{d \mathcal {Z}_B}{dt} = g_{Cl,eB}\left( V_B- E_{Cl,B}\right) - g_{Cl,AB}\left( (V_A- E_{Cl,A})-(V_B- E_{Cl,B})\right) , \end{aligned}$$

38h $$\begin{aligned}&\frac{ d \mathcal {W}_B }{ dt } = \nu _{eB}(\mathcal {O}_B-\mathcal {O}_e) - \nu _{AB} (\mathcal {O}_A-\mathcal {O}_B) . \end{aligned}$$

In terms of the new variables, the osmolarity in compartments A and B becomes:

$$\begin{aligned} \mathcal {O}_A=\frac{F}{\mathcal {W}_A}(\mathcal {X}_A+\mathcal {Y}_A+\mathcal {Z}_A+FX_A), \; \mathcal {O}_B=\frac{F}{\mathcal {W}_B}(\mathcal {X}_B+\mathcal {Y}_B+\mathcal {Z}_B+FX_B). \end{aligned}$$

The Nernst potential for each ion in the compartments *A*, *B* can be expressed as:

$$\begin{aligned} E_{\text {ion},A}=\frac{RT}{F{z_{ion}}}\ln \left( \frac{\mathcal {W}_A[\text {ion}]_e}{\mathcal {ION}_A}\right) , \; E_{\text {ion},B}=\frac{RT}{F{z_{ion}}}\ln \left( \frac{\mathcal {W}_B[\text {ion}]_e}{\mathcal {ION}_B}\right) , \end{aligned}$$

where $\mathcal {ION}=\mathcal {X},\mathcal {Y}, \mathcal {Z}$ for *Na*, *K*, *Cl*, respectively. Linearizing (38) around the steady state (28) yields an 8×8 Jacobian matrix *M*, which can be written in block form as

39 $$\begin{aligned} M = \begin{bmatrix}M^{A} & M^{dB} \\ M^{dA} & M^{B} \end{bmatrix}. \end{aligned}$$

The matrix $M^{A}$ is a 4×4 matrix defined by

$${\begin{bmatrix} -G_{A}^{Na}(\frac{1}{C_{m,A}}+\frac{RT}{F}\frac{1}{\mathcal {X}_A}) & -G_{A}^{Na}\frac{1}{C_{m,A}} & G_{A}^{Na}\frac{1}{C_{m,A}} & G_{A}^{Na}\frac{RT}{F} \frac{1}{\mathcal {W}_A} \\ - G_{A}^{K}\frac{1}{C_{m,A}} & -G_{A}^{K}(\frac{1}{C_{m,A}}+\frac{RT}{F}\frac{1}{\mathcal {Y}_A}) & G_{A}^{K}\frac{1}{C_{m,A}} & G_{A}^{K}\frac{RT}{F}\frac{1}{\mathcal {W}_A} \\ G_{A}^{Cl}\frac{1}{C_{m,A}} & G_{A}^{Cl}\frac{1}{C_{m,A}} & -G_{A}^{Cl}(\frac{1}{C_{m,A}}+\frac{RT}{F}\frac{1}{\mathcal {Z}_A}) & G_{A}^{Cl}\frac{RT}{F}\frac{1}{\mathcal {W}_A} \\ \nu _A\frac{F}{\mathcal {W}_A} & \nu _A\frac{F}{\mathcal {W}_A} & \nu _A\frac{F}{\mathcal {W}_A} & -\nu _A\sigma _A\frac{F}{\mathcal {W}_A^2} \end{bmatrix}} $$

where $$G_{A}^{Na}=g_{Na,eA}+g_{Na,AB}$$, $$G_{A}^{K}=g_{K,eA}+g_{K,AB}$$, $$G_{A}^{Cl}=g_{Cl,eA}+g_{Cl,AB}$$, $$\nu _A=\nu _{eA}+\nu _{AB}$$, and $$\sigma _A=\mathcal {X}_A+\mathcal {Y}_A+\mathcal {Z}_A+FX_A$$.

Also, $M^{dB}$ is a 4×4 matrix defined by

$$ \begin{bmatrix} g_{Na,AB}(\frac{1}{C_{m,B}} + \frac{RT}{F}\frac{1}{\mathcal {X}_B}) & g_{Na,AB}\frac{1}{C_{m,B}} & -g_{Na,AB}\frac{1}{C_{m,B}} & -g_{Na,AB}\frac{RT}{F}\frac{1}{\mathcal {W}_B} \\ g_{K,AB}\frac{1}{C_{m,B}} & g_{K,AB}(\frac{1}{C_{m,B}}+\frac{RT}{F}\frac{1}{\mathcal {Y}_B}) & -g_{K,AB}\frac{1}{C_{m,B}} & -g_{K,AB}\frac{RT}{F}\frac{1}{\mathcal {W}_B} \\ -g_{Cl,AB}\frac{1}{C_{m,B}} & -g_{Cl,AB}\frac{1}{C_{m,B}} & g_{Cl,AB}(\frac{1}{C_{m,B}}+\frac{RT}{F}\frac{1}{\mathcal {Z}_B}) & -g_{Cl,AB}\frac{RT}{F}\frac{1}{\mathcal {W}_B} \\ -\nu _{AB}\frac{F}{\mathcal {W}_B} & -\nu _{AB}\frac{F}{\mathcal {W}_B} & -\nu _{AB}\frac{F}{\mathcal {W}_B} & \nu _{AB}\sigma _B\frac{F}{\mathcal {W}_B^2} \end{bmatrix} , $$

Interchanging *A* and *B* in the above arguments for $M^{A}$ and $M^{dB}$ yields the corresponding arguments for $M^{B}$ and $M^{dA}$, respectively. Note that $g_{ion,BA} = g_{ion,AB}$ and $\nu _{AB} = \nu _{BA}$.

**Fig. 5:**
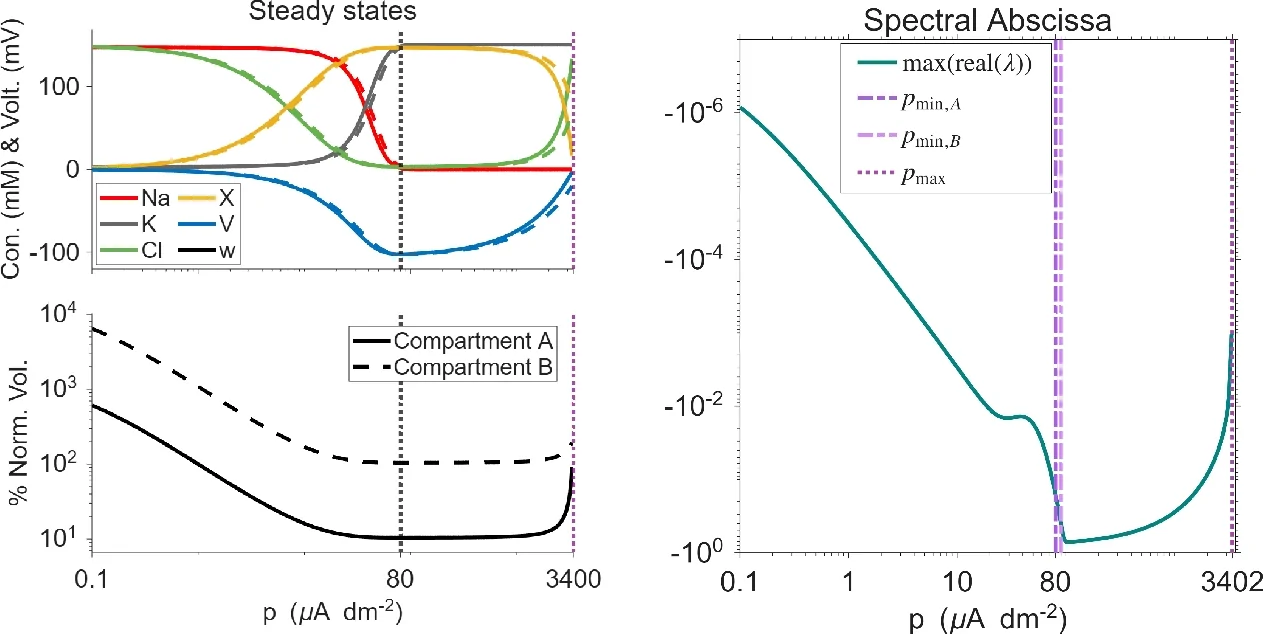
Steady state values of the ABp system (Equation (28)) and corresponding largest eigenvalues are plotted as functions of the pump rate p. As shown in the right panel, all eigenvalues are negative for any given p, indicating that the steady states are locally stable. The vertical lines mark $p_{\min ,j}$ and $p_{\max }$, corresponding to the pump rates at which the steady state volume reaches its minimum and to the largest value of p for which steady states exist, respectively. Both set of axes are shown on a logarithmic scale. All other parameters are fixed at their default values given in Tables 1–4 (color figure online)

For now, we fix all parameters at their default values (Tables 1–4) and vary only the pump rate p from near zero to $p_{\max } :=\max \{p_{\max ,A},\,p_{\max ,B}\},$ the full range over which steady states exist (See Figure 5, left panel). Later, in Section 5, we will analyze local stability as the other parameters vary. For any fixed p, the right panel of Figure 5 shows the largest eigenvalue among the eight real eigenvalues of *M*. For all values of p between 0 and $p_{max}$, the eigenvalues are real and negative, indicating local stability. Since the Jacobian *M* is nearly singular throughout this range, we compute its eigenvalues symbolically using variable-precision arithmetic to reduce round-off errors caused by ill-conditioning (Stor et al. 2015).

### Special ABp systems

In what follows, we examine two biologically relevant classes of ABp systems: The Koefoed-Johnsen-Ussing (KJU) model of epithelial transport and intracellular organelles. The KJU case is represented by an ABp model with $\overline{g}_{Na,eA}= \overline{g}_{K,AB}= 0$. The intracellular organelles case corresponds to an AB model, obtained from the ABp framework by removing the permeable paracellular pathway, i.e., by setting $\overline{g}_{\text {ion},eB}=0, \nu _{eB} = 0$. These two cases parallel classical studies of epithelial transport and pump-leak cell volume regulation (Tosteson and Hoffman 1960), and they provide mathematically tractable model systems for our analysis.

**KJU model.** The foundational model for epithelial transport is the Koefoed-Johnsen-Ussing (KJU) model, which was extended by ( Keener and Sneyd (2009), Section 2.8.2) to incorporate impermeant intracellular solutes, which were not included in the original formulation. In the KJU model, sodium conductance on the basolateral membrane and potassium conductance on the apical surface are set to zero ($\overline{g}_{Na,eA}=\overline{g}_{K,AB}= 0$), reflecting physiological asymmetries. In this configuration, sodium and water are transported from the apical to the basolateral side and can flow continuously at steady state. A related single-cell epithelial model that includes a paracellular pathway was developed by Weinstein and collaborators Strieter et al. (1990), capturing volume regulation via volume-activated chloride permeability. In contrast, here we consider the KJU model only in the context of a static epithelial vesicle.

In what follows, we compare the steady-state values of an KJU system with those of a general ABp system. To ensure that Assumption 1 holds, we require $\overline{g}_{K,eA},\,\overline{g}_{K,eB}> 0$ and $\overline{g}_{Na,AB},\,\overline{g}_{Na,eB}> 0$, which guarantees that Equation (25) is satisfied. We further assume that at least two of $\nu _{eA}$, $\nu _{AB}$, and $\nu _{eB}$ are nonzero. Under these conditions, the steady states of the KJU system—like those of the ABp system—are given explicitly by Equation (28) and therefore depend on several model parameters. Since $\overline{g}_{K,AB}= 0$, we have $G_{K,A} = 1/\overline{g}_{K,eA}$ and $G_{K,B} = 0$. Therefore, even though $\overline{g}_{K,eB}> 0$, the steady-state values of compartments *A* and *B* depend only on the potassium conductance along the basolateral membrane. In particular, no $\hbox {K}^+$ leaks into or out of compartment *B*.

In the KJU system, $f_{B,{eA}}$ has a unique negative root, so $f_{B,{eA}}(p) < 0$ for all p≥0, that is, $p_{\max ,B}= \infty$. In this case, we set $p_{\max }= p_{\max ,A}< \infty$. Furthermore, since $\overline{g}_{K,AB}= 0$, we obtain $p_{\min ,B}= \infty$, and since $\overline{g}_{Na,eA}= 0$, $p_{\min ,A}$ becomes much smaller (approximately ten times smaller) than $p_{\min ,A}$ in a general ABp system. This indicates that the KJU system requires less energy to minimize $w_A^{ss}$ compared to the ABp system. This observation is consistent with the results shown in Figures 9 and 11, where small $\overline{g}_{Na,eA}$ decreases the volume steady states in the presence of a weak pump mechanism (i.e., small p).

**Fig. 6:**
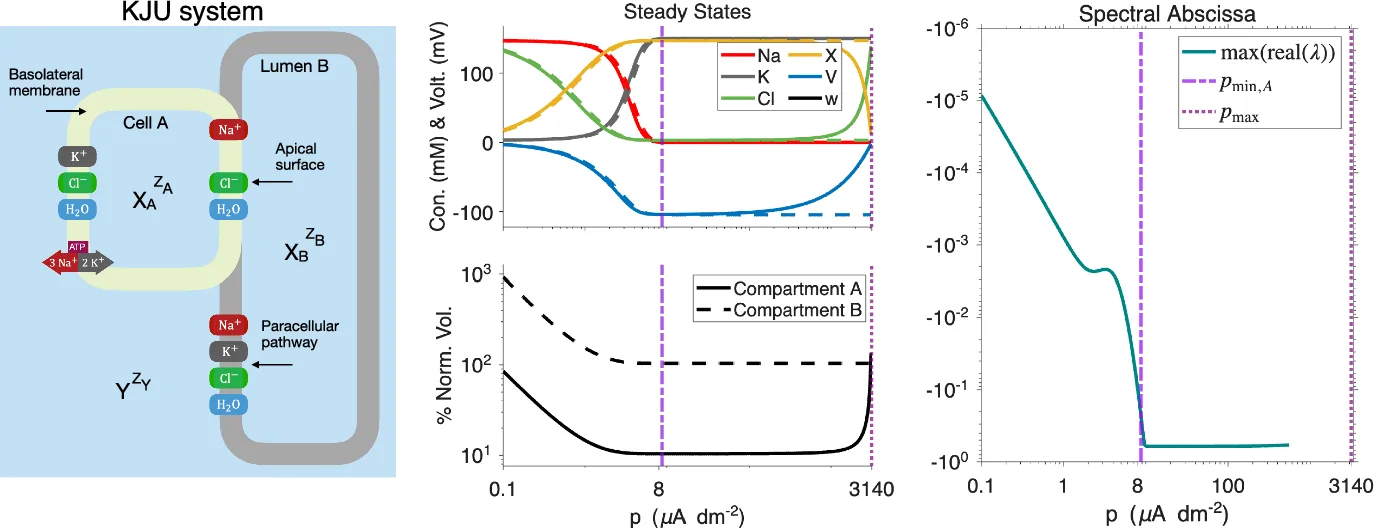
KJU system. The schematic diagram is shown, and the steady-state values together with the corresponding spectral abscissa are plotted as functions of the pump rate p. Conductances are fixed at $\overline{g}_{Na,eA}=\overline{g}_{K,AB}=0$ with remaining parameters fixed at their default value (color figure online)

Figure 6 depicts a schematic diagram for the KJU system (left), in which the sodium channels on the basolateral membrane and the potassium channels on the apical surface are removed. The steady states are shown as functions of the pump rate p for $p< p_{\max ,A}$. The value of $p_{\min ,A}$ is indicated by a vertical dashed line and is very small. Finally, the spectral abscissa (the maximum real part among the eigenvalues of the Jacobian at steady state) is plotted as a function of p, remaining negative and thereby reflecting the stability of the steady states.

**Intracellular organelles**. Intracellular organelles such as the nucleus, mitochondria, and endoplasmic reticulum are enclosed by lipid bilayer membranes that are topologically separate from the plasma membrane and that partition their interiors from the cytoplasm. Because these organelles are surrounded by selective membranes and exchange ions with the cytoplasm, their volumes, ionic concentrations, and voltage can be modeled using pump-leak equations. In our framework, the organelle interior plays the role of compartment *B* in an AB system with no paracellular pathway, that is, with $\overline{g}_{\text {ion},eB}=0, \nu _{eB} = 0$.

In what follows, we consider an AB system, a special case of the ABp system without a paracellular pathway. Since in an AB system we have $\overline{g}_{\text {ion},eB}=0, \nu _{eB} = 0$, the existence of a steady state requires that all other ion conductances are positive and that $\nu _{eA} > 0$, $\nu _{AB} > 0$.

A simple calculation shows that when $\overline{g}_{\text {ion},eB}= 0$, we have $G_{\text {ion},A}= G_{\text {ion},B}= 1/g_{\text {ion},eA}$, and therefore $\mathcal {C}_A = \mathcal {C}_B$ (see Equation (23)). The steady states depend only on two ion conductances, $\overline{g}_{Na,eA}$ and $\overline{g}_{K,eA}$. Increasing $\overline{g}_{Na,eA}$ increases the steady state volume, while increasing $\overline{g}_{K,eA}$ decreases it. Hence, if $z_{A}= z_{B}$, the two compartments admit identical steady states (except for volume, which differs by a factor $X_A/X_B$). We conclude that the two-compartment AB system effectively behaves like a single cell. Thus, similar to the equilibrium values of Equation (11), the only parameters that distinguish the two compartments are $z_{A}$ and $z_{B}$.

**Fig. 7:**
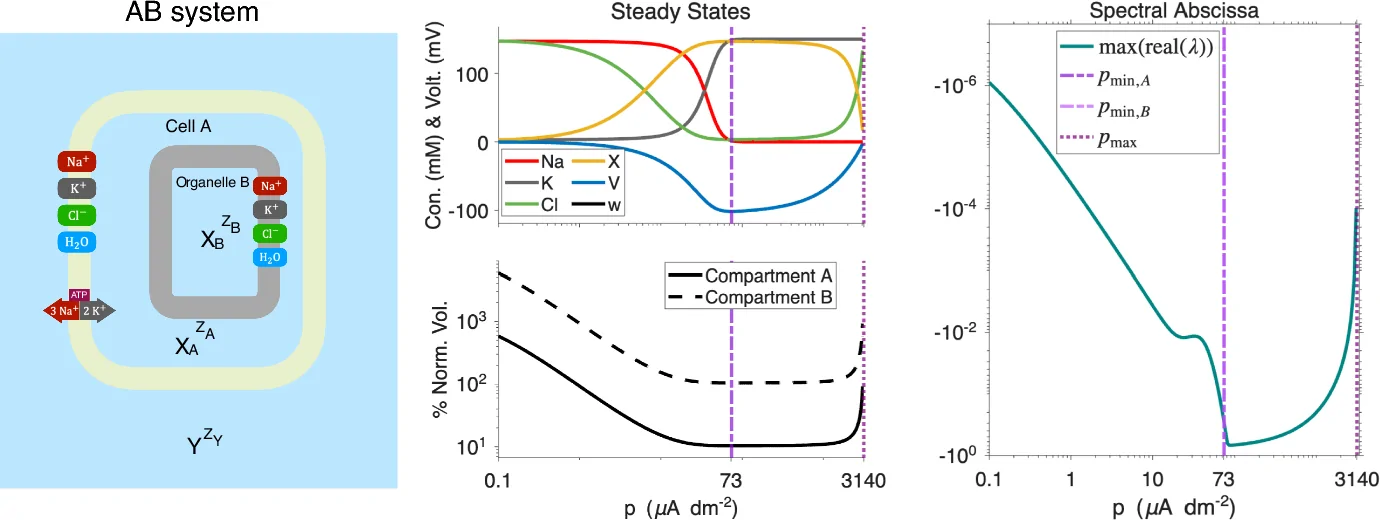
AB system. The schematic diagram is shown, and the steady-state values together with the corresponding spectral abscissa are plotted as functions of the pump rate p. Assigning $X_B=X_A/10$ will make the volume of compartment B a tenth of A’s (color figure online)

Figure 7 depicts a schematic diagram for the AB system (left), in which the channels on the paracellular pathway are removed. The steady states are shown as functions of the pump rate p. The value of $p_{\min ,A}$ is indicated by a vertical dashed line and is very small. Finally, the spectral abscissa is plotted as a function of p, remaining negative and thereby reflecting the stability of the steady states.

## Effects of Biophysical Parameters on ABp System

In Equation (28) of Section 4, we derived explicit expressions for the steady states of the coupled PLEs for general ABp systems. In Figure 5, these steady states are plotted as functions of p for a representative set of parameter values (our default values). However, as shown in Table 5 in the appendix, model parameters can vary across cell types. Because the steady-state expressions depend nonlinearly on many parameters, it is difficult to assess parameter influence by direct inspection. To address this challenge and to ensure that our predictions remain valid over a broader parameter range—and thus across different cell types—we perform a sensitivity analysis in the following three subsections. Specifically, we (1) characterize the steady-state behavior of ABp systems, with emphasis on mechanisms that stabilize compartmental volumes; (2) examine the robustness of these behaviors under parameter variations; and (3) identify the parameter regimes in which the ABp system maintains homeostasis.

### Key mechanisms that stabilize the volumes in ABp systems

We already saw that the NKA pump is a key mechanism that stabilizes cell and lumen volumes, and our results in the ABp system are consistent with those shown before in single-compartment models (Aminzare and Kay 2024; Mori 2012; Keener and Sneyd 2009; Fraser and Huang 2007). In this section, we use variance-based global sensitivity analysis (VBGSA) (Saltelli et al. 2004, 2008) to identify the key model parameters that exhibit compensatory mechanisms for maintaining volume stabilization when the NKA pump activity is reduced.

VBGSA quantifies how uncertainty in parameters contributes to the variance of an output of interest (here, a steady state value of cell volume). It is a global, rather than local, sensitivity analysis, as the perturbation of multiple parameters is performed simultaneously (Jansen 1999; Saltelli et al. 2008). For a given outcome value, the total variance is decomposed into contributions from each parameter or group of parameters and compared to the total variance in the output. This decomposition can be summarized by Sobol indices, which are dimensionless numbers ranging from 0 to 1. A Sobol index close to 1 indicates that the parameter has a strong influence on the steady state, while an index close to 0 indicates a weak influence. Sobol indices partition output variance into first- and total-order indices which indicate the contributions of each parameter (Saltelli et al. 2010; Dela et al. 2022).

To apply VBGSA for our model and compute the Sobol indices, we consider the interaction among nine parameters: the pump rate; sodium and potassium conductances across the basolateral membrane, apical membrane, and paracellular pathway; extracellular concentrations–primarily the combination of sodium and chloride–and the temperature. Note that chloride conductances do not appear in the steady-state formulas, although they are essential for the operation of the PLM. Moreover, to preserve electroneutrality and positive osmolarity, not all extracellular concentrations can be varied simultaneously. All other model parameters are fixed at their default values.

To vary $[Na]_e$ and $[Cl]_e$ in a charge-balanced manner, we introduce the reparameterization

40 $$\begin{aligned} [NaCl]_e= [Na]_e+ [Cl]_e . \end{aligned}$$

This additional parameter enables us to simultaneously vary $[Na]_e$ and $[Cl]_e$ while maintaining electroneutrality since the charges of the two ions cancel each other out. The perturbed parameters are $[Na]_e= [Na]_e^{\text {default}} + [NaCl]_e$ and $[Cl]_e= [Cl]_e^{\text {default}} + [NaCl]_e$.

**Fig. 8:**
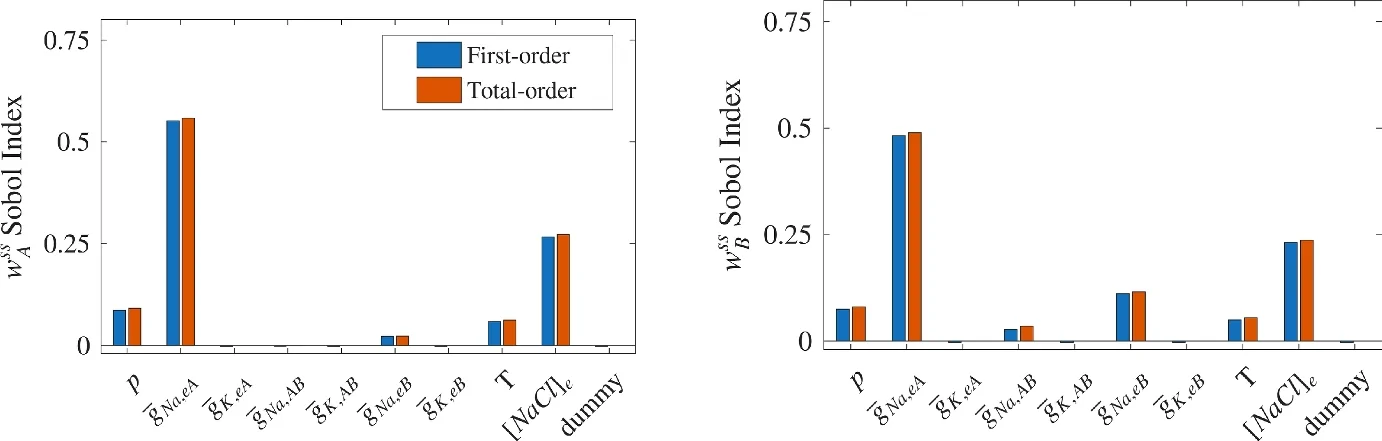
First- and total-order Sobol indices of the steady-state volumes of compartments *A* (left) and *B* (right) are shown as the nine parameters are varied as described in (46). The Sobol indices indicate that p, $\overline{g}_{Na,eA}$, *T*, and $[NaCl]_e$ contribute most to the variance in $w_A^{ss}$, with the largest contribution arising from $\overline{g}_{Na,eA}$ (highest bars) when p is small (using default value 1 μA $\hbox {dm}^{-2}$). Similarly, the Sobol indices of $w_B^{ss}$ show that these same parameters are the most influential, with additional contributions from $\overline{g}_{Na,AB}$ and substantially larger contributions from $\overline{g}_{Na,eB}$ (color figure online)

In Figure 8, we use VBGSA to compute the first-order (blue bars) and total-order (orange bar) Sobol indices of the steady-state volumes of compartments A (left) and B (right) as the following nine parameters vary:

$$p,\; \overline{g}_{Na,eA},\; \overline{g}_{K,eA},\; \overline{g}_{Na,AB},\; \overline{g}_{K,AB},\; \overline{g}_{Na,eB},\; \overline{g}_{K,eB},\; T,\; [NaCl]_e. $$

More details about the range of these parameters and computing the Sobol indices are described in the Appendix A.4.

As Figure 8 depicts, the Sobol indices show that the variance in the steady-state volume of compartment A is primarily attributable to variations in p, $\overline{g}_{Na,eA}$, $[NaCl]_e$, and *T*. Similarly, the variance in the steady-state volume of compartment B is mainly attributable to changes in p, $\overline{g}_{Na,eA}$, $\overline{g}_{Na,AB}$, $\overline{g}_{Na,eB}$, $[NaCl]_e$, and *T*.

Although the Sobol index diagram identifies which parameters influence the steady-state volume, it does not indicate whether these changes increase or decrease the volume. To address this, we perturb only two parameters at a time and plot the resulting steady-state values as heat maps.

Another reason to study the heat maps, rather than relying solely on the Sobol indices, is that the Sobol indices in Figure 8 are computed over a restricted range of the pump rate, namely p∈[0.1,10] (μA $\hbox {dm}^{-2}$). This restriction is imposed because, for larger values of p, variations in the pump rate account for a disproportionately large fraction of the output variance—particularly in volume-related quantities—thereby masking the contributions of other parameters in the Sobol index analysis. In contrast, for the heat-map analysis that follows, we consider a broader range of pump rates, p∈[0.1,150] (μA $\hbox {dm}^{-2}$), in order to ensure physiologically meaningful system behavior. In this regime, the model yields ion concentrations and membrane potentials within acceptable ranges for compartments *A* and *B*, specifically sodium concentrations between 1–30 mM, potassium concentrations between 50–100 mM, chloride concentrations between 1–30 mM, and membrane potentials between -100 and -40 mV.

Motivated by the Sobol sensitivity analysis, we first investigate whether modulating basolateral sodium conductance can serve as an additional or alternative mechanism for modulating volume, particularly when the pump rate is low. For example, *in vivo* experiments have shown that tetrodotoxin and saxitoxin act on voltage-gated sodium channels to attenuate sodium conductance (Narahashi et al. 1964; Chrachri and Williamson 1997; Maltsev et al. 1998). We vary the basolateral sodium conductance $\overline{g}_{Na,eA}$ between $10^{-2}$ to $10^{2}$ mS $\hbox {dm}^{-2}$, which is a physiologically relevant range (Weinstein 2012, 2015).

**Fig. 9:**
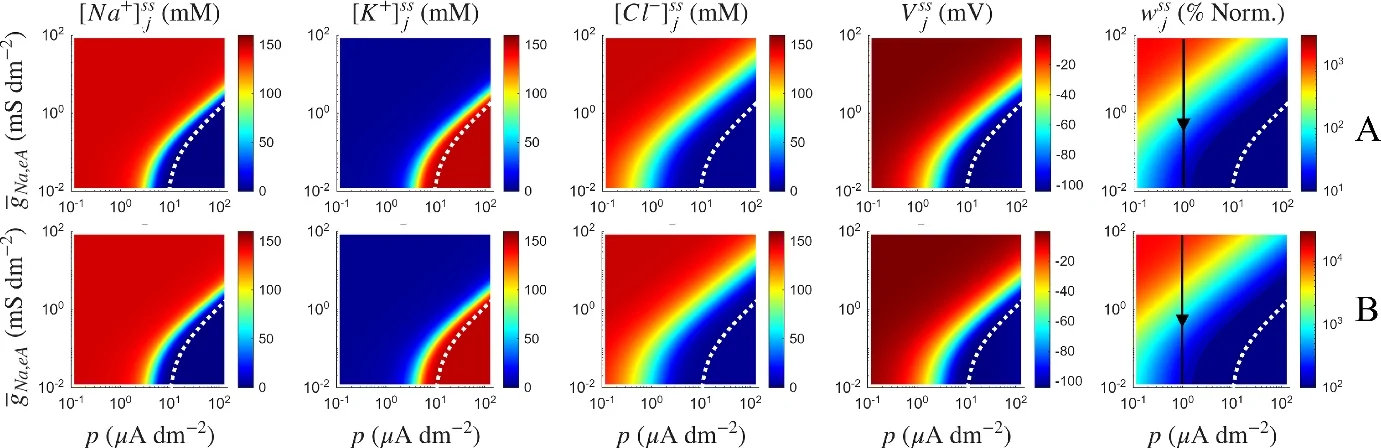
Steady-state values of compartment *A* (top) and *B* (bottom) as functions of p, $\overline{g}_{Na,eA}$, shown as heat maps. The black vertical line indicates that when p is small, decreasing $\overline{g}_{Na,eA}$ decreases $w_A^{ss}$ and $w_B^{ss}$. The dashed white curve traces the locus $p=p_{\min ,A}(\overline{g}_{Na,eA})$(top) and $p=p_{\min ,B}(\overline{g}_{Na,eA})$ (bottom) at which $w_A^{ss}$ and $w_B^{ss}$ reach their respective minima—computed from Equation (36) (color figure online)

Figure 9 depicts the steady-state values as heat maps on p∈[0.1,150] (in μA $\hbox {dm}^{-2}$) and $\overline{g}_{Na,eA}\in [0.01, 100]$ (in mS $\hbox {dm}^{-2}$). The left four columns show the changes in intracellular ion concentrations and voltage, and the right column shows the corresponding volume.

The right column of Figure 9 shows that decreasing $\overline{g}_{Na,eA}$ decreases the steady-state volume of compartments *A* and *B* (indicated by the vertical black line and arrow). This suggests that blocking the sodium channel on the basolateral membrane could assist the pump mechanism in reducing the volume of the ABp system. This result is consistent with our previous work on a single cell (Aminzare and Kay 2024), where we showed that reducing the sodium conductance lowers $p_{\min }$, the pump-rate value that minimizes the steady-state volume. The left columns of Figure 9 shows that, for small pump rates, changes in sodium conductance do not appreciably alter the concentrations or membrane potential; a larger pump rate is required to observe such effects.

Similar heat map analysis show that for a fixed small p, decreasing $\overline{g}_{Na,AB}$ (respectively, $\overline{g}_{Na,eB}$) slightly decreases the volume of *A* but substantially increases (respectively, decreases) the volume of *B*, as indicated by the Sobol indices corresponding to $\overline{g}_{Na,AB}$. For more details, see Figures 19–20 in Section A.5 of Appendix.

In summary, all sodium conductances can be used to control the volume of either or both compartments. Physiologically, such effects of sodium conductances make sense because limiting sodium entry reduces intracellular sodium accumulation, thereby lowering the osmotic gradient that draws water into the cell. This not only decreases the need for compensatory ion pumping but also prevents osmotic swelling, especially under stress or pathological conditions. Experimental studies support this prediction: in epithelial and astrocytic cells, amiloride-sensitive ENaC channels on the apical surface have been shown to mediate swelling-activated sodium conductance that can be pharmacologically suppressed to reduce volume in the cell (Kizer et al. 1997); and knockout mice lacking claudin-2 had a depressed sodium flux across the paracellular pathway and showed a decrease in reabsorption of water into the lumenal space (Muto et al. 2010). These studies confirm that sodium conductance is a key regulator of cell volume and a potential therapeutic target.

Furthermore, the Sobol index diagram predicts that perturbing the extracellular sodium and chloride concentrations–i.e., perturbing $[NaCl]_e$ as defined in (40)–and the temperature *T* affect the steady-state cell volume. The corresponding heat map analysis shows that increasing $[NaCl]_e$ and decreasing *T* lead to a decrease in the steady-state cell volume, especially when the pump rate is low. See the Appendix for further discussion of the effects of extracellular sodium–chloride concentration and the temperature.

### Robustness of multiple volume control mechanisms

Robustness is the ability of a system to maintain its functions against perturbations (Kitano 2004). In biological systems, robustness is essential for survival in changing environments, such as the homeostatic mechanisms that maintain intracellular volume despite fluctuations in extracellular conditions (Alberts et al. 2022). In Figure 5, we showed that an ABp system relies on the NKA pump as its primary mechanism for controlling ion concentrations, voltage, and volume. Furthermore, in Section 5.1, we identified alternative mechanisms that can stabilize volume when the pump is weak. In particular, we observed that sodium conductances, extracellular concentrations, and temperature can modulate the volume. Thus far, we have varied only one (in Figure 5) or two (in Section 5.1) of these parameters at a time to examine their effects. However, in nature, simultaneous fluctuations in multiple parameters are inevitable. In this section, we study the robustness of these behaviors, in the sense of determining whether they persist under perturbations to multiple parameters.

**Fig. 10:**
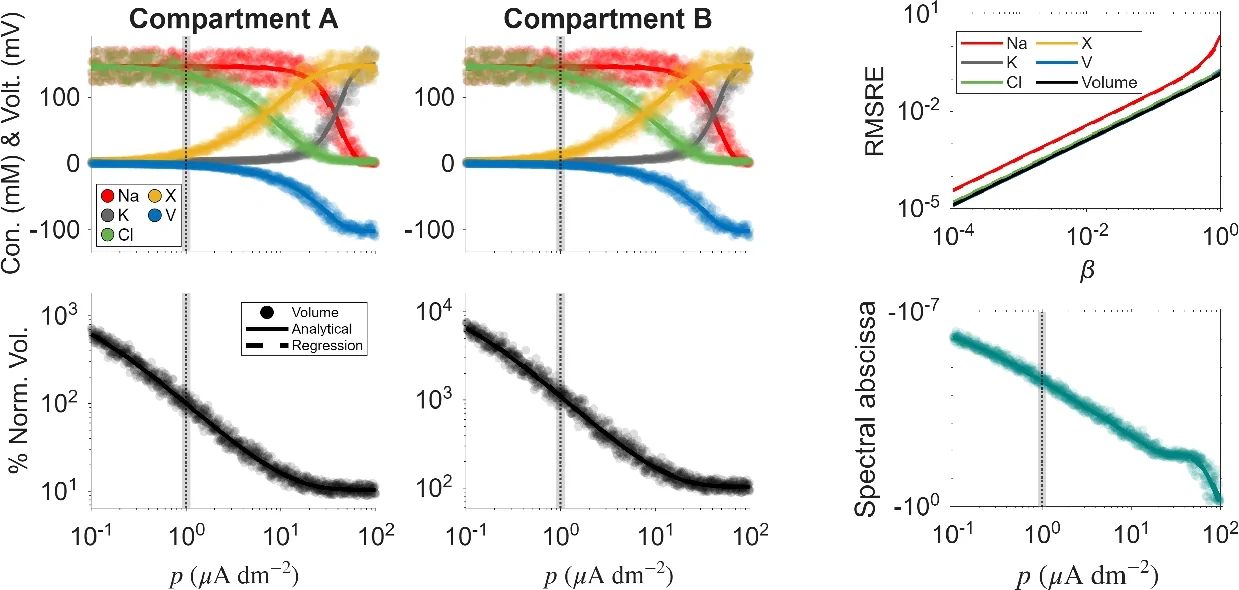
(Left & Middle.) Steady-state values of the ABp system are shown as functions of the pump rate p∈(0,100): solid curves correspond to the default parameter values listed in Tables 2 and 3; scattered points correspond to 1000 parameter vectors of dimension 9 sampled via LHS; and dashed curves represent GP regression fits to the scattered data. **(Bottom Right.)** Spectral abscissa of the Jacobian evaluated at the steady states shown in the left and middle panels. **(Top Right.)** Root mean square relative error (RMSRE), defined in Equation (41), for the state variables in compartments *A* (solid) and *B* (dashed), plotted as a function of the scaling factor β, which increases the magnitude of the perturbations in $\overline{g}_{\text {ion},(\cdot )}$, *T*, and $[NaCl]_e$. GP regressions overlap with analytical steady states (color figure online)

**Fig. 11:**
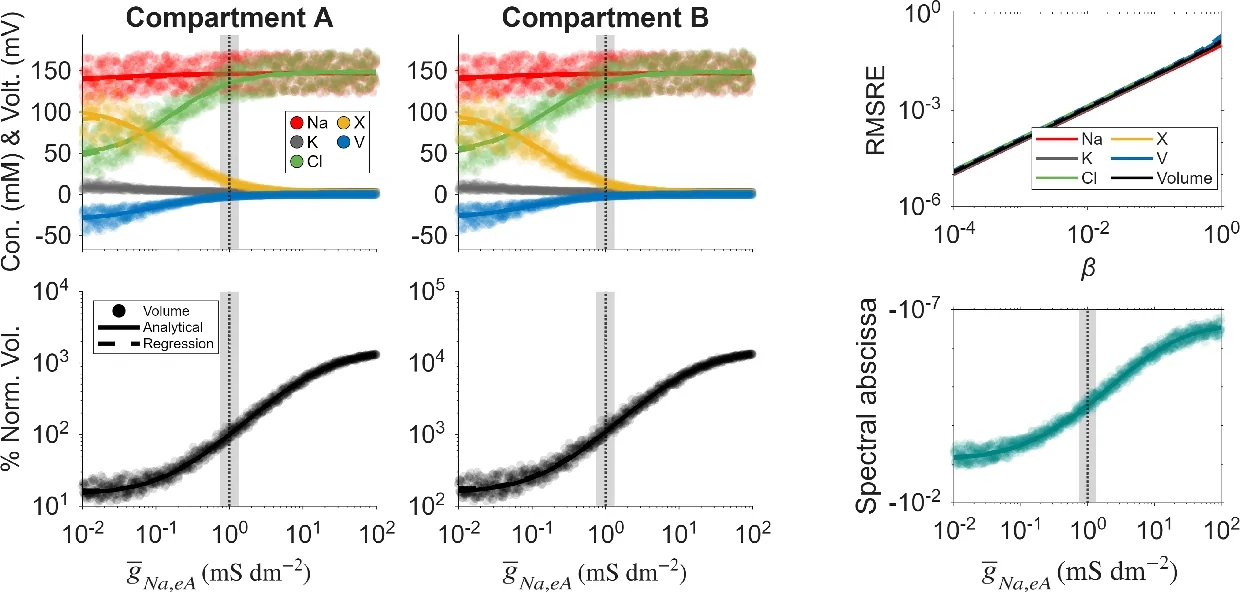
Steady-state values of the ABp system and corresponding spectral abscissa are shown as functions of the sodium conductance $\overline{g}_{Na,eA}$. The gray shaded vertical bands mark the perturbation described in (46) around the default value (dashed vertical line) of $\overline{g}_{Na,eA}$. The RMSRE between scattered points and analytically computed steady states show that the error increases as the perturbation factor β increases. GP regressions overlap with analytical steady states (color figure online)

**Fig. 12:**
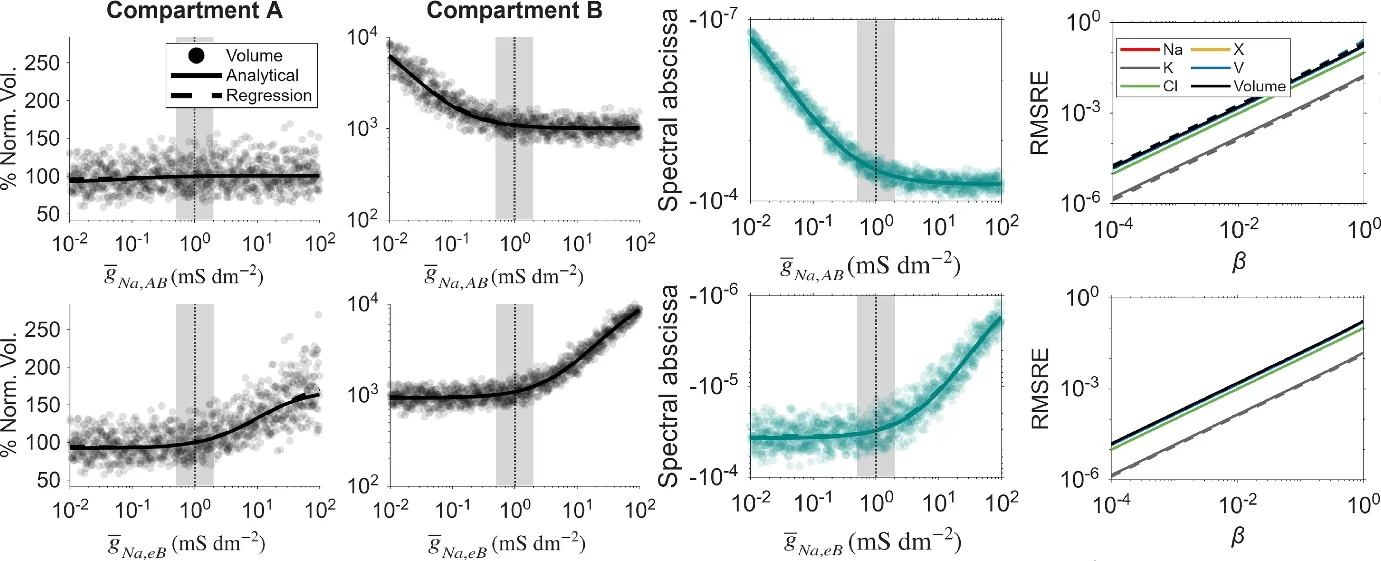
A comparison of the steady-state values of the volumes and corresponding spectral abscissa are shown as functions of $\overline{g}_{Na,AB}$ (top) or $\overline{g}_{Na,eB}$ (bottom). Varying these conductances has little effect on concentrations and voltage (not shown). GP regressions overlap with analytical steady states (color figure online)

**Fig. 13:**
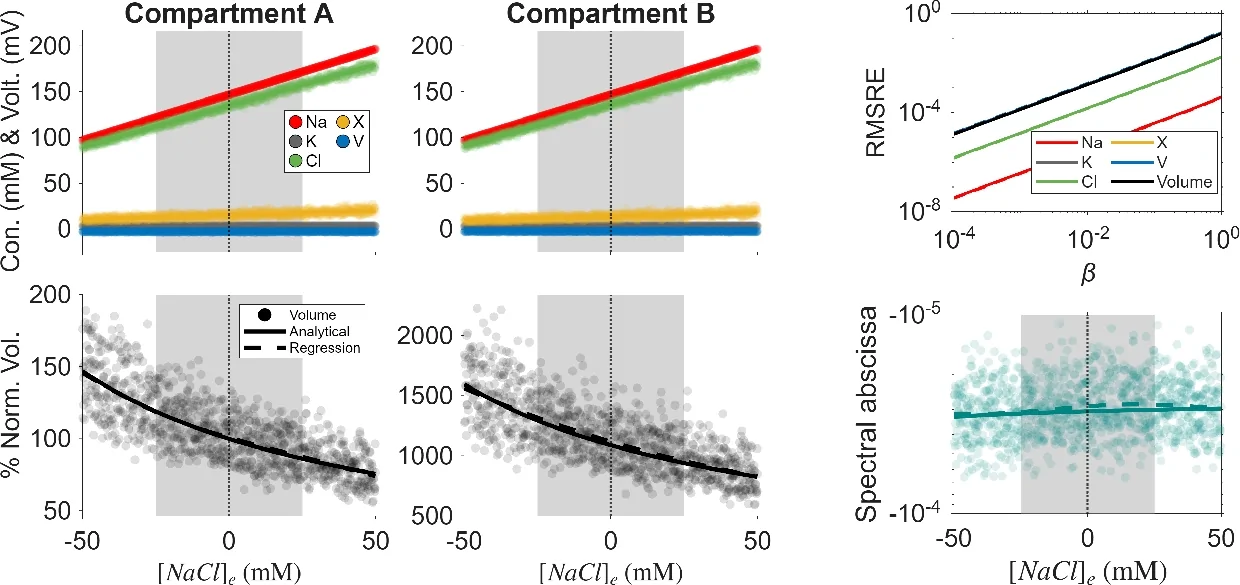
Steady-state values of the ABp system and corresponding spectral abscissa are shown as functions of $[NaCl]_e$ and the corresponding RMSRE are shown as functions of the perturbation factor β. GP regressions overlap with analytical steady states (color figure online)

Similar to Figure 8, we simultaneously perturb $\overline{g}_{\text {ion},(\cdot )}$, $[NaCl]_e$, and *T* (as described in Equation (47) in the appendix), and plot the analytically computed steady-state values (from Equations (27)–(28)) of the ABp system as functions of p (Figure 10), $\overline{g}_{Na,(\cdot )}$ (Figures 11 and 12), and $[NaCl]_e$ (Figure 13) to assess the robustness of the behaviors observed in Section 5.1.

In these figures, the solid curves and scatter points represent the steady-state values and the spectral abscissa of the Jacobian (from Equations (39)), evaluated at the *default* and *perturbed* parameter values, respectively. There are 1000 scatter points.

As described above, we perturb nine parameters. Specifically, we consider a 9-dimensional hypercube centered around the default parameter values and use Latin hypercube sampling (LHS) to generate 1000 samples from this hypercube. For each sampled parameter set, we compute the steady states and the corresponding spectral abscissa, producing the scatter points shown in the figures.

We plot two additional curves: One is the root mean square relative error (RMSRE) (Despotovic et al. 2016; Webber et al. 2017; Göçken et al. 2016), which measures the average relative deviation of the perturbed steady states from those corresponding to the default parameter values, and is defined as

41 $$\begin{aligned} \text {RMSRE} = \sqrt{\mathbb {E} \left[ \left( \frac{{\boldsymbol{y}} - {\boldsymbol{o}}}{{\boldsymbol{o}}}\right) ^2\right] }. \end{aligned}$$

where ${\boldsymbol{y}}$ and ${\boldsymbol{o}}$ denote the steady-state values (or the corresponding largest eigenvalues) for the perturbed and default parameters, respectively. Plotting the RMSRE as a function of β (the perturbation factor) illustrates how rapidly the deviation from the default steady state grows as the perturbation window widens

Finally, we plot the Gaussian process (GP) regressions (Rasmussen and Williams 2006) fitted to the scatter points, which serve as surrogate models for the perturbed data. The GP regressions are shown as dashed curves that largely overlap with the solid curves corresponding to the default parameters.

For details of the above procedures, see Appendix  A.4.

All figures confirm that although the RMSRE increases as the perturbation factor β grows (top-right panels), the steady-state values of the ABp system still exist and remain locally stable under the considered parameter fluctuations (green curves in the bottom-right panels). Overall, the system behavior remains robust, as explained next.

Figure 10, in comparison with Figure 5, confirms that in the presence of parameter fluctuations, increasing p continues to decrease the steady-state volumes.

Furthermore, Figures 11 and 12, when compared with the black arrows in Figures 9, 19, and 20 (from the appendix), confirm that under relatively weak NKA pump activity and in the presence of appropriate parameter fluctuations, decreasing the sodium conductances on the basolateral membrane and the paracellular pathway ($\overline{g}_{Na,eA}$ and $\overline{g}_{Na,eB}$) continues to decrease the steady-state volumes. Similarly, decreasing the sodium conductance on the apical surface ($g_{Na,AB}$) increases the steady-state volume in compartment *B* while having little decreasing effect in compartment *A*.

We perform the same robustness analysis for intermediate and high pump rates (p=40,90 μA $\hbox {dm}^{-2}$), where the ion concentrations reach more physiologically realistic values—i.e., high potassium, low sodium and chloride concentrations, and low voltage—and observe similar behavior. The results are provided in Appendix A.6.

Finally, Figure 13 examines the robustness of the effects of extracellular concentrations on ABp system behavior, as previously discussed (and as shown in Figure 21 in the appendix). Under relatively weak NKA pump ($p\in [0.9,\,1.\overline{1}]$) and in the presence of parameter fluctuations, increasing the extracellular sodium and chloride concentrations (i.e., increasing $[NaCl]_e$) continues to decrease the steady-state volume, (consistent with the black arrow shown in the right panel of Figure 21).

### Parameters that preserve homeostasis

**Fig. 14:**
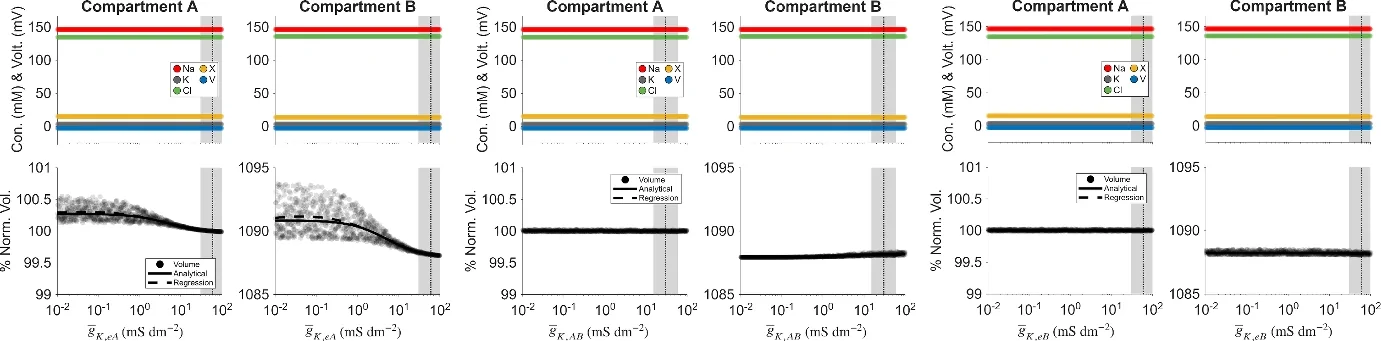
Homeostasis of steady states with respect to potassium conductances. Steady-state responses of the ABp system under simultaneous perturbations of the potassium conductances $\overline{g}_{K,eA}$, $\overline{g}_{K,AB}$, and $\overline{g}_{K,eB}$. In each panel, one conductance is varied across its full tested range while the remaining two are perturbed within their sampling intervals (color figure online)

In the sensitivity analysis, we observed that the potassium conductances $\overline{g}_{K,eA}$, $\overline{g}_{K,AB}$, and $\overline{g}_{K,eB}$ contribute negligibly to the variance of the steady-state values; see the short bars associated with these parameters in Figure 8. This insensitivity is further illustrated in Figure 14, where all three potassium conductances are perturbed simultaneously and the resulting steady states are projected onto each conductance (left-to-right panels). Across the tested parameter ranges, the scatter points remain tightly clustered around the analytical steady-state curve, indicating that the steady state is effectively unchanged under combined fluctuations in $\overline{g}_{K,(\cdot )}$. Thus, in contrast to sodium conductances, the ABp system exhibits an insensitivity to variations of the potassium conductance. Likewise, primarily in single-cell settings, Weinstein predicted that modulation of basolateral $\hbox {K}^+$ permeability does not, per se, significantly blunt challenges to cell volume (Weinstein 1999) and it is also shown by Edwards and Layton (2017).

Chloride conductances do not appear in the closed-form steady state expressions derived in Section 4, so varying $\overline{g}_{Cl,eA}$, $\overline{g}_{Cl,AB}$, or $\overline{g}_{Cl,eB}$ does not alter the steady state ion concentrations, voltages, or volumes. In this sense, for fixed values of the remaining parameters, the ABp steady state is invariant under sufficiently small fluctuations in $\overline{g}_{Cl,(\cdot )}$, and the system is homeostatic with respect to these conductances. The chloride conductances do, however, enter the Jacobian (39) and therefore influence the eigenvalues and transient dynamics. Assumption 1 ensures that no more than one of $\overline{g}_{Cl,eA}$, $\overline{g}_{Cl,AB}$, and $\overline{g}_{Cl,eB}$ is fixed at zero. In addition, within the admissible parameter set, perturbations in the chloride conductances change the magnitude of the spectral abscissa but not its sign, so local stability is preserved. For this reason, we hold $\overline{g}_{Cl,(\cdot )}$ fixed at their default values in our sensitivity and robustness analyses. See Section A.7. of the Appendix for further discussion on the transient effects of chloride conductances.

## Active Two-Compartment Systems: NKA Pump on Apical Surface

In a less common configuration, the NKA pump mechanism can be found on the apical surface, interfacing compartments *A* and *B*, without a corresponding pump mechanism on the basolateral membrane. This occurs in the epithelial cells of the choroid plexus, which secrete cerebrospinal fluid into the ventricles of the brain (Brown et al. 2004). We extend the PLEs to cases where the passive and active transporters generate ionic gradients along the apical surface, while the basolateral membrane solely possesses ion channels. In Equations (2)–(6), we let ${{\textbf {p}}}_{{{\textbf {Na,eA}}}}= 0, {{\textbf {p}}}_{{{\textbf {K,eA}}}}= 0, {{\textbf {p}}}_{{{\textbf {Na,AB}}}}=-\gamma _{Na} \, p\, a_{AB}, {{\textbf {p}}}_{{{\textbf {K,AB}}}}= \gamma _{K} \, p\, a_{AB}.$

The NKA pump hydrolyzes ATP in compartment *A*, expending energy to pump $\gamma _{Na}$ Na^+^ ions into compartment *B* for every $\gamma _K$ K^+^ pumped from *B* into *A*. The ionic channels and paracellular pathway facilitate the passive transport of ions and water between regions. From our steady state equations in Section 4 and numerical solutions of the ODE system (2)–(6), we compare the effects of pump rates on ionic flux at steady state and in a time series.

**Existence of steady states. ** The form of the steady states for the PLEs with an apical surface pump mechanism is very similar to that of the PLEs with a basolateral membrane pump mechanism derived in Section 4.1, with the following modifications. In Lemma 2 and Proposition 2, replace $p\, a_{eA}$ by $p\, a_{AB}$, and replace $G_{\text {ion},A}$ and $G_{\text {ion},B}$ by

42a $$\begin{aligned}&G_{\text {ion},A}^{(2)}= \dfrac{g_{\text {ion},eB}}{g_{\text {ion},eA} g_{\text {ion},AB} + g_{\text {ion},eA} g_{\text {ion},eB} + g_{\text {ion},AB} g_{\text {ion},eB}} \end{aligned}$$

42b $$\begin{aligned}&G_{\text {ion},B}^{(2)}= \dfrac{-g_{\text {ion},eA}}{g_{\text {ion},eA} g_{\text {ion},AB} + g_{\text {ion},eA} g_{\text {ion},eB} + g_{\text {ion},AB} g_{\text {ion},eB}} . \end{aligned}$$

**Fig. 15:**
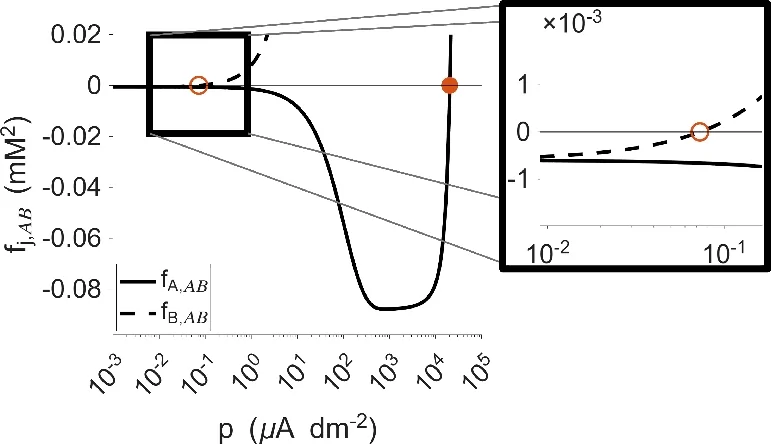
The admissible range of p is computed numerically by plotting $f_{j,{AB}}(p):= 4 \, \mathcal {C}_{j}^2(p) - \mathcal {O}_e^2$ and identifying the interval $(0, p_{\max })$ where $f_{j,{AB}}(p) < 0$. In the zoomed-in panel, the open circle denotes the root of $f_{B,{AB}}$ (color figure online)

**The possible range of the pump rate. ** In this case, unlike the ABp system with the NKA pump located on the basolateral membrane (see Figure 5), the ABp system becomes highly sensitive to small changes in the pump rate p. In particular, the steady state of compartment *B* exists only over a restricted range of pump values, namely for $p< p_{\max ,B}$, where $p_{\max ,B}$ is substantially smaller than $p_{\max ,A}$ for compartment *A*. See Equation (35) and Figure 15 for a comparison of $p_{\max ,B}$ and $p_{\max ,A}$. Consequently, the existence of a stable steady state with non-negative concentrations and volume is confined to a relatively narrow range of p.

**Fig. 16:**
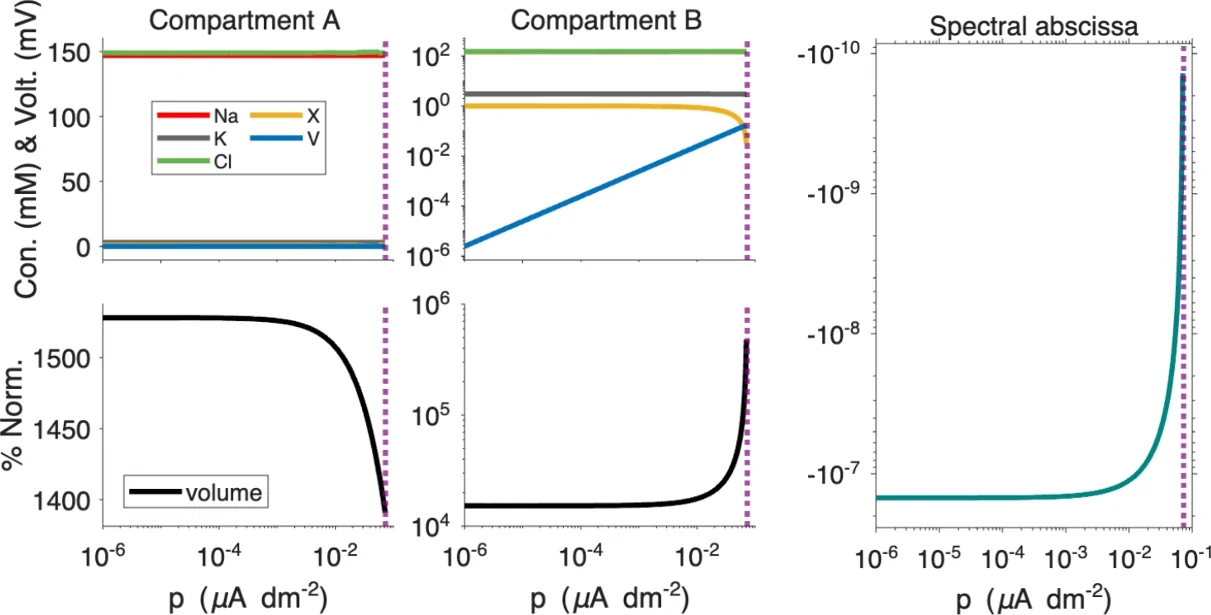
ABp system with the NKA pump on the apical surface. Steady states and their corresponding spectral abscissae are plotted as functions of the pump rate p. The purple vertical line marks $p_{\max ,B}$, the largest value of p for which a steady state with non-negative volume exists. $[Na]_B^{ss}$ and $[Cl]_B^{ss}$ remain approximately the same over this range of p and are plotted on top of one another in the middle panel (color figure online)

**Explicit form of steady states and their stability. ** Figure 16 shows the steady-state values of an ABp system against p, where the pump is located on the apical surface and $p\le p_{\max ,B}$. All concentrations and volumes remain non-negative across this range since the condition $f_{j,{AB}}(p):= 4 \, \mathcal {C}_{j}^2(p) - \mathcal {O}_e^2<0$ remains valid.

**Fig. 17:**
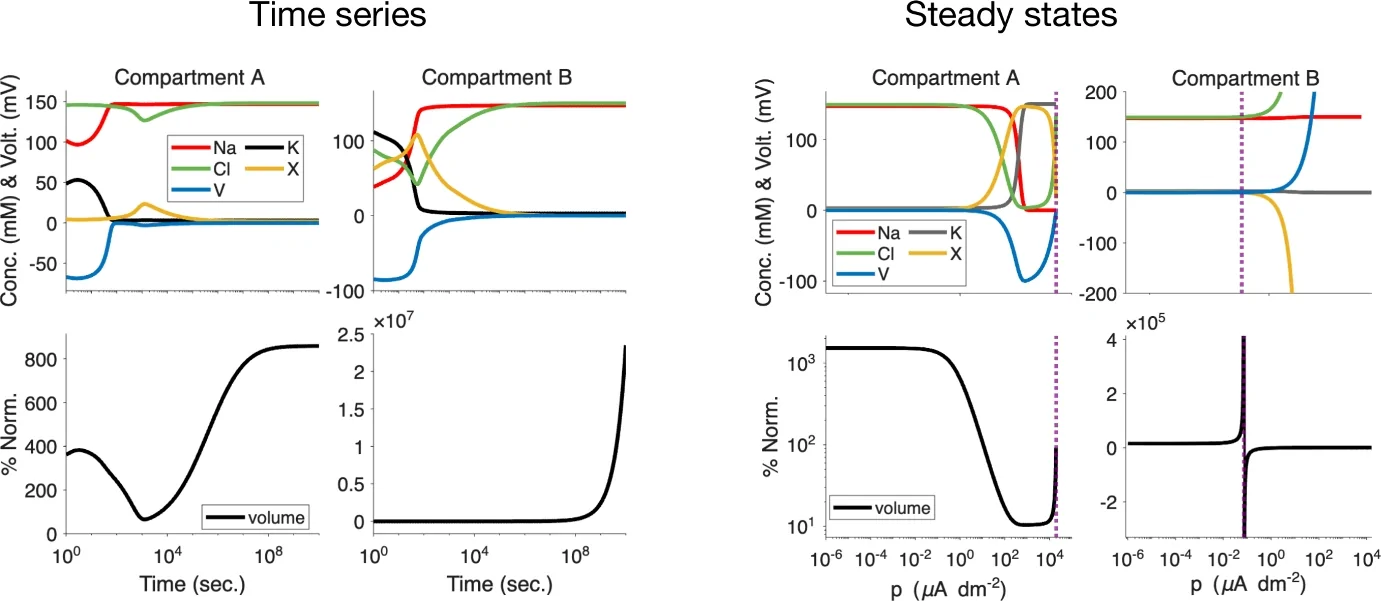
(Left) Time series of a numerically computed solution of the coupled PLEs with $p= 2 \times p_{\max ,B}$. (Right) Steady state values are plotted as functions of the pump rate p for a larger range of p, $0<p<p_{\max ,A}$ which violates the condition for existence of steady states with non-negative volumes. So on the right-bottom panel, $w_B^{ss}$ increases to ∞ for $p<p_{\max ,B}$ and becomes negative for $p>p_{\max ,B}$. This corresponds to epithelial transport in the time series where $w_B(t)$ grows without bound (color figure online)

Although steady states of the ABp system with non-negative concentrations and volumes for both compartments *A* and *B* exist only for $p< p_{\max ,B}$, compartment *A* remains stable for $p_{\max ,B}< p< p_{\max ,A}$, while compartment *B* exhibits unbounded growth. Figure 17 (left panels) shows the time series of a numerically computed solution of the coupled PLEs with $p= 2 \times p_{\max ,B}$. As illustrated, all variables in compartments *A* and *B* approach finite steady-state values except for the volume of *B*, which diverges. This behavior persists for all $p< p_{\max ,A}$, as demonstrated in the right panel of Figure 17. Note that $w_B^{ss}$ appears as a negative value for $p> p_{\max ,B}$ because the condition $f_{B,{AB}} < 0$ is violated; in practice, the volume does not become negative but instead grows without bound.

**A note on ion flows in an ABp system at steady state.** For an ABp system with the NKA pump located on the apical surface, the ionic fluxes across the three interfaces are

43a $$\begin{aligned}&\Phi _{\text {ion},eA}= z_{\text {ion}}\, g_{\text {ion},eA}\left( V_A- E_{\text {ion},A}\right) , \end{aligned}$$

43b $$\begin{aligned}&\Phi _{\text {ion},AB}= - z_{\text {ion}}\, g_{\text {ion},AB}\left[ \left( V_A- E_{\text {ion},A}\right) - \left( V_B- E_{\text {ion},B}\right) \right] + {{\textbf {p}}}_{{{\textbf {ion,AB}}}}, \end{aligned}$$

43c $$\begin{aligned}&\Phi _{\text {ion},eB}= - z_{\text {ion}}\, g_{\text {ion},eB}\left( V_B- E_{\text {ion},B}\right) , \end{aligned}$$

where ${{\textbf {p}}}_{{{\textbf {Na,AB}}}}=-\gamma _{Na} \, p\, a_{AB}$, ${{\textbf {p}}}_{{{\textbf {K,AB}}}}= \gamma _{K} \, p\, a_{AB},$ and ${{\textbf {p}}}_{{{\textbf {Cl,AB}}}}=0.$ As in Equation (37), $\Phi _{\text {ion},{\cdot }}$ denotes the active flux (i.e., in the presence of the pump).

Using the steady-state expressions in Equation (28), together with $G_{\text {ion},A}^{(2)}$ and $G_{\text {ion},B}^{(2)}$ from Equation (42), one verifies that the absolute values of the total ion—and water—fluxes are equal across the basolateral, apical, and paracellular pathways for $p< p_{\max ,B}$; that is,

$$ \Phi _{\text {ion},eA}^{ss} = \Phi _{\text {ion},AB}^{ss} = \Phi _{\text {ion},eB}^{ss}. $$

**Fig. 18:**
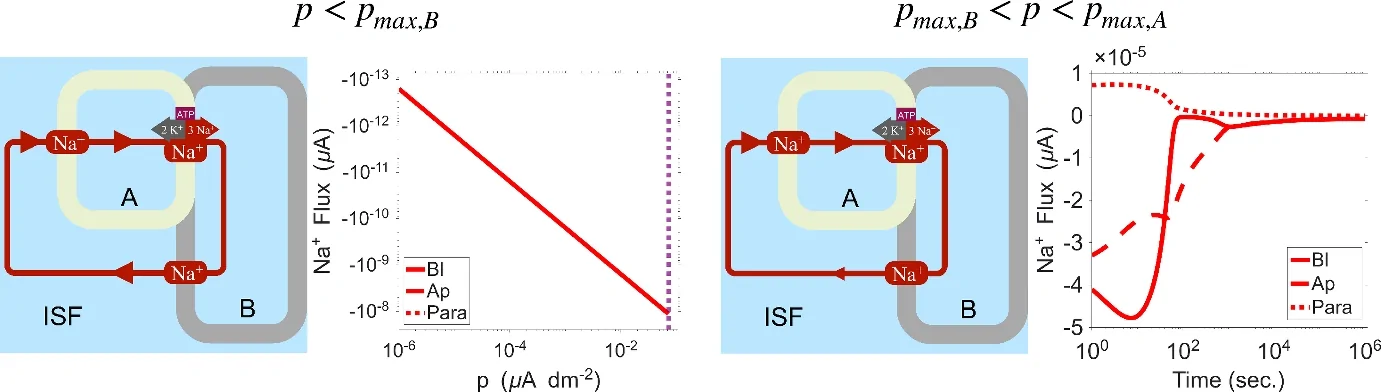
Sodium transport across the membranes at steady state for an ABp system with the NKA pump on the apical surface. The schematic diagrams illustrate the clockwise sodium transport loop. For $p< p_{\max ,B}$, the total sodium fluxes across the three membranes are equal at steady state (second panel from left). For $p> p_{\max ,B}$, the flux through the paracellular pathway becomes smaller than the apical and basolateral fluxes (i.e., larger in the same direction as the fluxes through Ap and Bl), leading to water accumulation in compartment *B* and resulting in its instability (right panel) (color figure online)

**Fig. 19:**
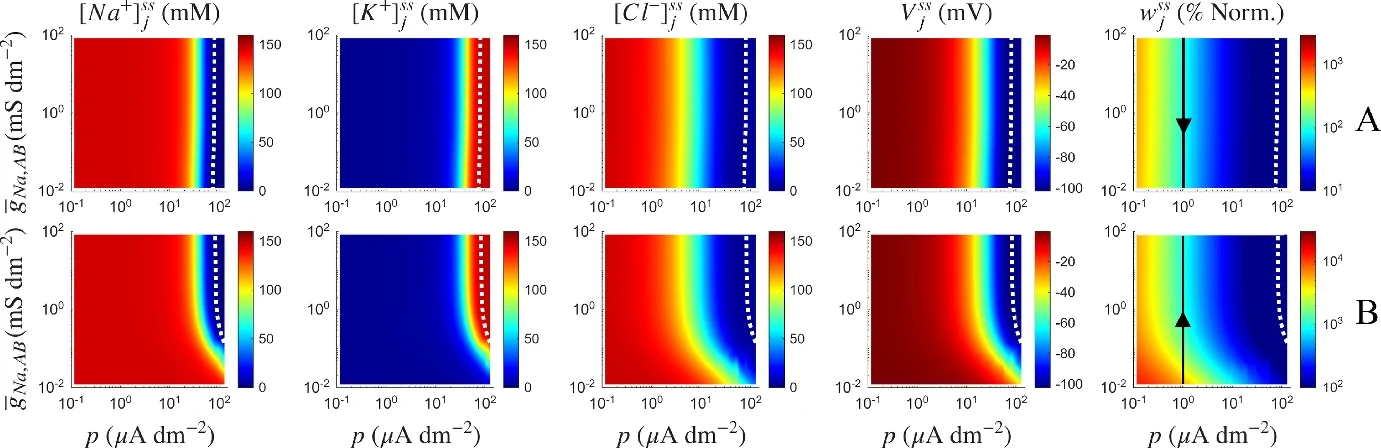
Steady-state values of compartments *A* (top) and *B* (bottom) as functions of p and $\overline{g}_{Na,AB}$, shown as heat maps. The black vertical line and arrow indicate that when p is small, decreasing $\overline{g}_{Na,AB}$ slightly decreases $w_A^{ss}$ while substantially increasing $w_B^{ss}$. The dashed white curve overlays the locus $p=p_{\min ,A}(\overline{g}_{Na,AB})$ in the top row and $p=p_{\min ,B}(\overline{g}_{Na,AB})$ in the bottom row, where $w_A^{ss}$ and $w_B^{ss}$ attain their respective minima—curves computed from Equation (36) (color figure online)

**Fig. 20:**
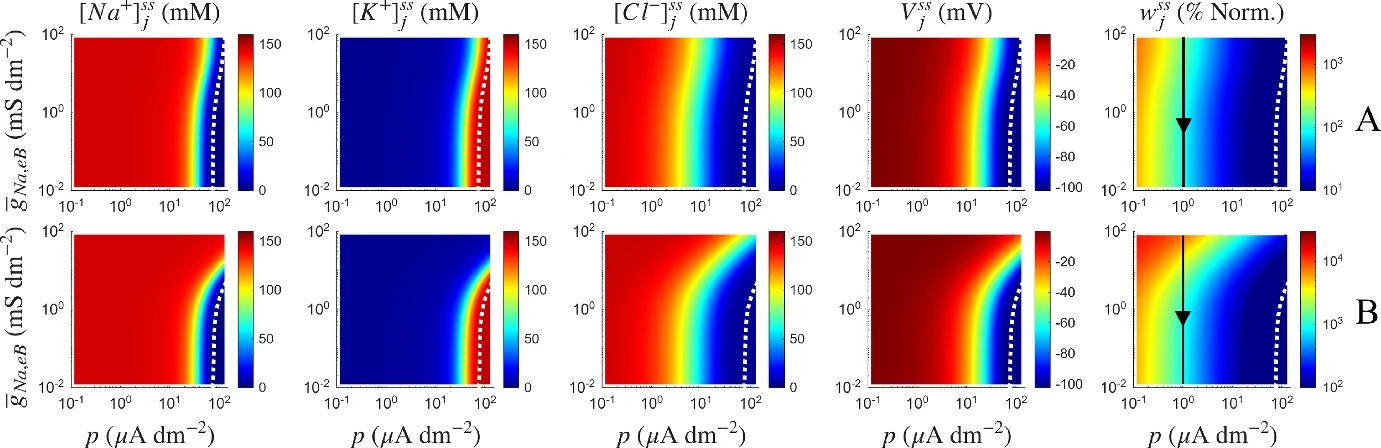
The layout follows that of Figure 19, with $\overline{g}_{Na,eB}$ replacing $\overline{g}_{Na,AB}$; for small p, decreasing $\overline{g}_{Na,eB}$ reduces $w_A^{ss}$ and $w_B^{ss}$, with a more pronounced effect on $w_B^{ss}$ (color figure online)

**Fig. 21:**
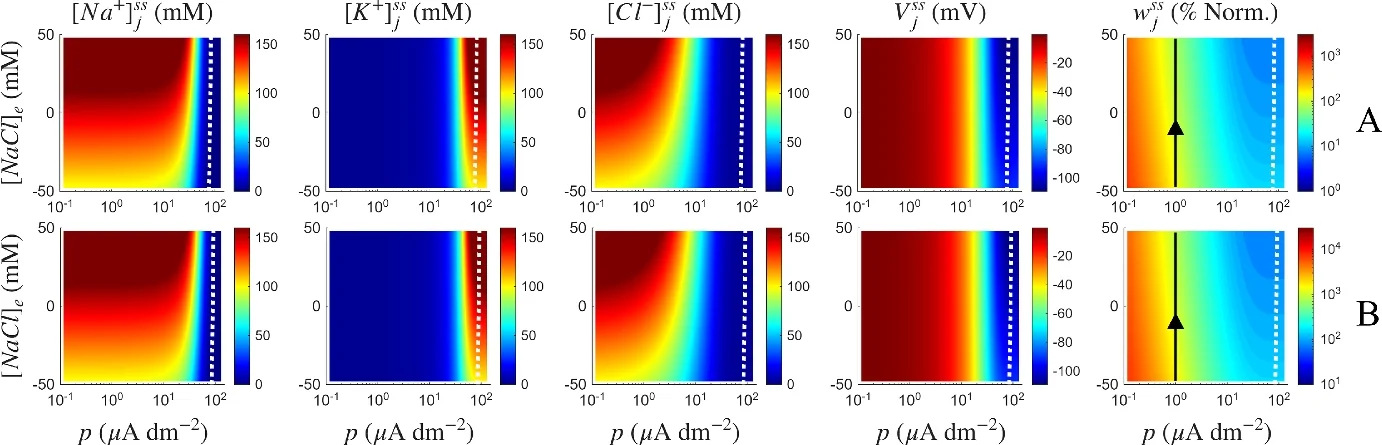
The layout of this figure follows that of Figure 19, with $[NaCl]_e$ replacing $\overline{g}_{Na,AB}$; for small p, increasing $[NaCl]_e$ decreases both $w_A^{ss}$ and $w_B^{ss}$ (color figure online)

As illustrated in the two left panels of Figure 18, sodium flow forms a clockwise loop in this configuration: sodium moves from compartment *B* to the ISF through the paracellular pathway, enters compartment *A* across the basolateral membrane, and returns to compartment *B* via the apical surface. The total fluxes across the three membranes are plotted in the second-left panel as functions of $p< p_{\max ,B}$. As p increases, the magnitude of the flux increases across all pathways while remaining equal, confirming conservation of flow at steady state. Unlike in Figure 4, the fluxes here are negative, indicating opposite flow directions.

In addition, in Figure 18 (right panels), we plot the time series of $\Phi _{\text {Na},eA}, \Phi _{\text {Na},AB}, \Phi _{\text {Na},eB}$ for some $p_{\max ,B}<p<p_{\max ,A}$ and observe that for large time *t*, i.e., at the steady state, the $\hbox {Na}^+$ fluxes become nearly constant and

$$\begin{aligned} \Phi _{\text {Na},eA}(t)\approx \Phi _{\text {Na},AB}(t)<\Phi _{\text {Na},eB}(t)<0. \end{aligned}$$

Thus, the magnitude of the paracellular pathway $\hbox {Na}^+$ flux $B\rightarrow \text {ISF}$ is smaller than the magnitude of the apical surface $\hbox {Na}^+$ flux A→B. This asymmetry produces a net $\hbox {Na}^+$ gain in compartment *B*, so $[Na]_B(t)$ increases. The resulting osmotic imbalance drives water into compartment *B* to compensate for the growing osmolarity, and $w_B(t)$ increases. The $\hbox {K}^+$ flux exhibits an analogous bottleneck in compartment *B*.

Apical NKA transport requires that the $\hbox {K}^+$ entering compartment A across the apical surface not be immediately lost across the basolateral membrane. If the basolateral $\hbox {K}^+$ conductance is too high, it dissipates the $\hbox {K}^+$ gradient generated by the pump and allows $\hbox {K}^+$ to leak out across the basolateral membrane. Keeping basolateral $\overline{g}_{K,eA}$ smaller maintains a high intracellular $\hbox {K}^+$ activity set by pump activity and allows $\hbox {K}^+$ recycling rather than $\hbox {K}^+$ loss (Zeuthen and Wright 1981; MacAulay et al. 2022). This is the same energy-conservation logic that underlies classic pump-leak models, but here it is applied to the less common apical NKA configuration. Consistent with this physiology, the mathematical ABp system admits a stable steady state when the apical $\hbox {K}^+$ conductance is large ($\overline{g}_{K,AB}$ high) and the basolateral $\hbox {K}^+$ conductance is small ($\overline{g}_{K,eA}$ low). A large $\overline{g}_{K,AB}$ provides the local $\hbox {K}^+$ recycling pathway needed to balance apical NKA current, while a small $\overline{g}_{K,eA}$ prevents the basolateral side from short-circuiting the apical pump and draining intracellular $\hbox {K}^+$. We note, however, that in more general settings, a high basolateral $\hbox {K}^+$ conductance does not necessarily dissipate the $\hbox {K}^+$ gradient if extracellular compartments are subject to inputs and outputs (e.g., fluid flow) that help maintain low extracellular $\hbox {K}^+$ concentrations.

Unlike the ABp system with basolateral NKA, where a sizable basolateral $\hbox {K}^+$ leak helps regulate the system, in the apical NKA case, the same leak becomes destabilizing because it competes directly with the pump for control of intracellular $\hbox {K}^+$ and volume. Not shown here, $\overline{g}_{K,AB}=300$ mS $\hbox {dm}^{-2}$ and $\overline{g}_{K,eA}=30$ mS $\hbox {dm}^{-2}$ are parameter values consistent with biological systems, in which $\hbox {K}^+$ conductance on the apical surface can be roughly 10 times larger than on the serosal surface (Zeuthen and Wright 1981).

## Discussion

This work provides a systematic extension of the classical pump–leak framework to a coupled, two-compartment system, referred to as the ABp system, demonstrating how spatial organization fundamentally alters the qualitative behavior of ionic concentrations, membrane potential, and volume. Our development of the ABp model extends the reach of the PLM to multicellular assemblies, in particular static epithelial vesicles, and serves as a prelude to the mathematical analysis of epithelial systems that transport fluid.

Despite the resulting 10-dimensional system of coupled algebraic–differential equations and the presence of many parameters, we derive explicit steady-state formulas for both passive and pump-driven active regimes under a constant pump mechanism and characterize their dependence on physiologically meaningful parameters. We further show that assuming a constant pump rate preserves the qualitative behavior of the system while rendering the analysis analytically accessible. Obtaining explicit expressions for steady states is especially important in this setting, as the system exhibits stiffness and strong parametric dependence, making purely numerical determination of steady states both challenging and unreliable across large regions of parameter space.

To assess local stability of these steady states, we derived the Jacobian matrix analytically. However, due to its dependence on multiple parameters, obtaining explicit analytical expressions for the eigenvalues is not practical. We therefore evaluate the eigenvalues numerically for a representative set of parameter values. Because stability depends on the chosen parameter set, we further examine robustness under parameter perturbations in Section 5.2. Previously Mori (2012) demonstrated how a free energy formulation of the PLM using Lyapunov functions could be used to study the global stability of the PLEs for a single cell. We leave the global asymptotic analysis of the ABp system for future studies.

While our analysis focuses on the characterization and local stability of stationary solutions, the transient behavior of trajectories remains an important aspect of the model. In particular, even when the stationary solutions are stable, trajectories may temporarily pass through physiologically unfavorable regimes before converging. A systematic investigation of such transient dynamics is beyond the scope of the present work, as our primary goal is to obtain analytical insight into steady-state structure and stability. Nevertheless, to provide some perspective on these effects, we include representative time-series in the Appendix (Section A.7), illustrating how solutions evolve under different parameter choices. These results suggest that, within the explored parameter ranges, trajectories converge without exhibiting extreme or unphysical excursions, although a more comprehensive analysis is warranted. A detailed study of transient behavior, including potential thresholds for adverse dynamics and their physiological implications, will be the subject of future work.

Although the steady states are derived explicitly, their expressions depend nonlinearly on numerous model parameters, making qualitative prediction of ABp system behavior challenging as parameters vary. To address this complexity, we perform a global sensitivity analysis that reveals a pronounced low-dimensional structure underlying the high-dimensional parameter space. Despite the large number of parameters, variance-based Sobol indices show that steady-state volume, voltage, and ionic concentrations are governed primarily by a small subset of parameters, notably pump strength, sodium conductances, and extracellular sodium concentration. This clear separation between dominant and negligible parameter directions suggests opportunities for systematic model reduction and provides a rigorous explanation for the robustness of volume regulation observed across wide parameter ranges.

The model developed here is for a rather specialized system, a stable epithelial vesicle. This model encompasses a wide range of natural biological structures as well as organoids. We initially developed the ABp to model a rather specialized biological structure, the scolopidium (or chordotonal organ), which serve as stretch receptors in insects (Field and Matheson 1998). Scolopidia have a specific glial cell, a scolopale cell that surrounds the dendrites of a sensory neuron. The scolopale cell creates a closed fluid filled compartment around the sensory dendrites with an elevated $\hbox {K}^+$ concentration. Another example of an ABp system is the scala media in the mammalian cochlea. In this case, a multilayer epithelium, the stria vascularis produces endolymph, which covers the apical surface of the organ of Corti and has a high $\hbox {K}^+$ concentration (Nin et al. 2012). Our model could also be of value in understanding the formation of pathological cysts such as occur in polycystic kidney disease (Bergmann et al. 2018).

It is worth noting that biomechanical models have been proposed to simulate the development of epithelial vesicles (Rejniak and Anderson 2008) and there is considerable interest in the formation of epithelial organoids in vitro (Lu et al. 2025; Pedersen et al. 1999).

In the ABp model it is possible for cyclic flows of $\hbox {Na}^+$ and $\hbox {K}^+$ to be generated by the operation of the NKA. Without an active $\hbox {Cl}^-$ transporter there are no net flows of this ion. In the model as implemented, no cyclic water flows can occur. However, when ions move through the channels they have an obligatory hydration shell, so the water can cycle, too. In the case of voltage-gated $\hbox {Na}^+$ channels, water accompanies the ion in its passage through the channel, whereas in the case of $\hbox {K}^+$ channels water is stripped from the ions (Roux 2017). If this is true for $\hbox {Na}^+$ leak channels, water will cycle through the ABp system. This is what is classically called electroosmosis (Finkelstein 1987). See Figure 4.

From a modeling perspective, although the ABp model introduced here provides a tractable extension of the classical PLEs and captures the essential dynamics of a cell coupled to a lumen—both reproducing known regulatory mechanisms and predicting new ones—it nevertheless has limitations that warrant further investigation. In particular, we model ionic currents using linear Ohm’s law, whereas a more biophysically detailed description could be obtained using the nonlinear Goldman–Hodgkin–Katz (GHK) formulation. Similarly, water transport is modeled using a Starling-type relation without explicit incorporation of hydrostatic effects, or ion-water interactions.

In addition, it remains to be shown rigorously under what conditions an ABp system provides an accurate reduced description of a multicompartment epithelial architecture of the form $A_1\dots A_NBp$. Extending the model to include ion cotransporters represents another important direction toward greater physiological realism.

From a mathematical perspective, while our analysis focuses on the existence, local stability, and robustness of steady states, the explicit formulas derived here lay the groundwork for deeper analytical investigations. The strong dependence of steady states on model parameters suggests the presence of rich bifurcation structures. A systematic bifurcation analysis, as well as the development of Lyapunov or free-energy methods adapted to two-compartment settings, may provide insight into global stability, transitions between stable regimes, and the onset of pathological behaviors such as luminal volume divergence.

Incorporating GHK-type nonlinearities will likely preclude closed-form expressions for steady states; consequently, the development of accurate and efficient numerical methods for computing steady states and their stability properties will be essential for advancing these models.

## Materials and Methods

All computations were performed using MATLAB, release 25.2.0.2998904 (R2025b) (The MathWorks, Inc., Natick, MA). A small capacitance allows rapid changes in voltage. In this case, the PLEs become “stiff,” and special numerical solvers are required to solve this system. In this work, for the nonlinear NKA models and to generate the time series shown in Figure 17 (left) and in the appendix, the system of equations was solved using the MATLAB stiff differential equation solvers ode15s and ode23tb (which produce similar results, differing only in computational speed depending on the parameter regime). To reduce the sensitivity of time series solutions to stiff solvers, we use the voltage formulation algebraically derived in Equation (3), which yields more numerically stable trajectories. The code is currently maintained in a private repository and is available upon reasonable request. The code will be made publicly available upon acceptance of the associated manuscript.

## Figures and Tables

**Fig. 22:**
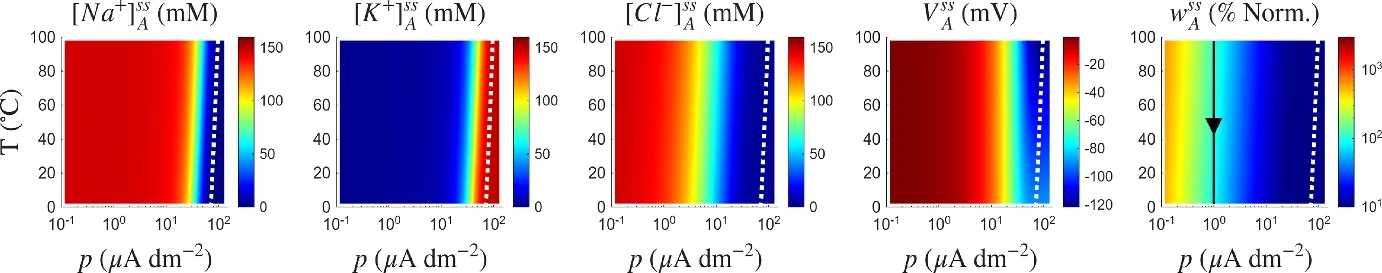
The layout of this figure follows that of Figure 19, with *T* replacing $\overline{g}_{Na,AB}$; for small p, decreasing *T* decreases $w_A^{ss}$. Similar trends hold for $w_B^{ss}$ (not shown) (color figure online)

**Fig. 23:**
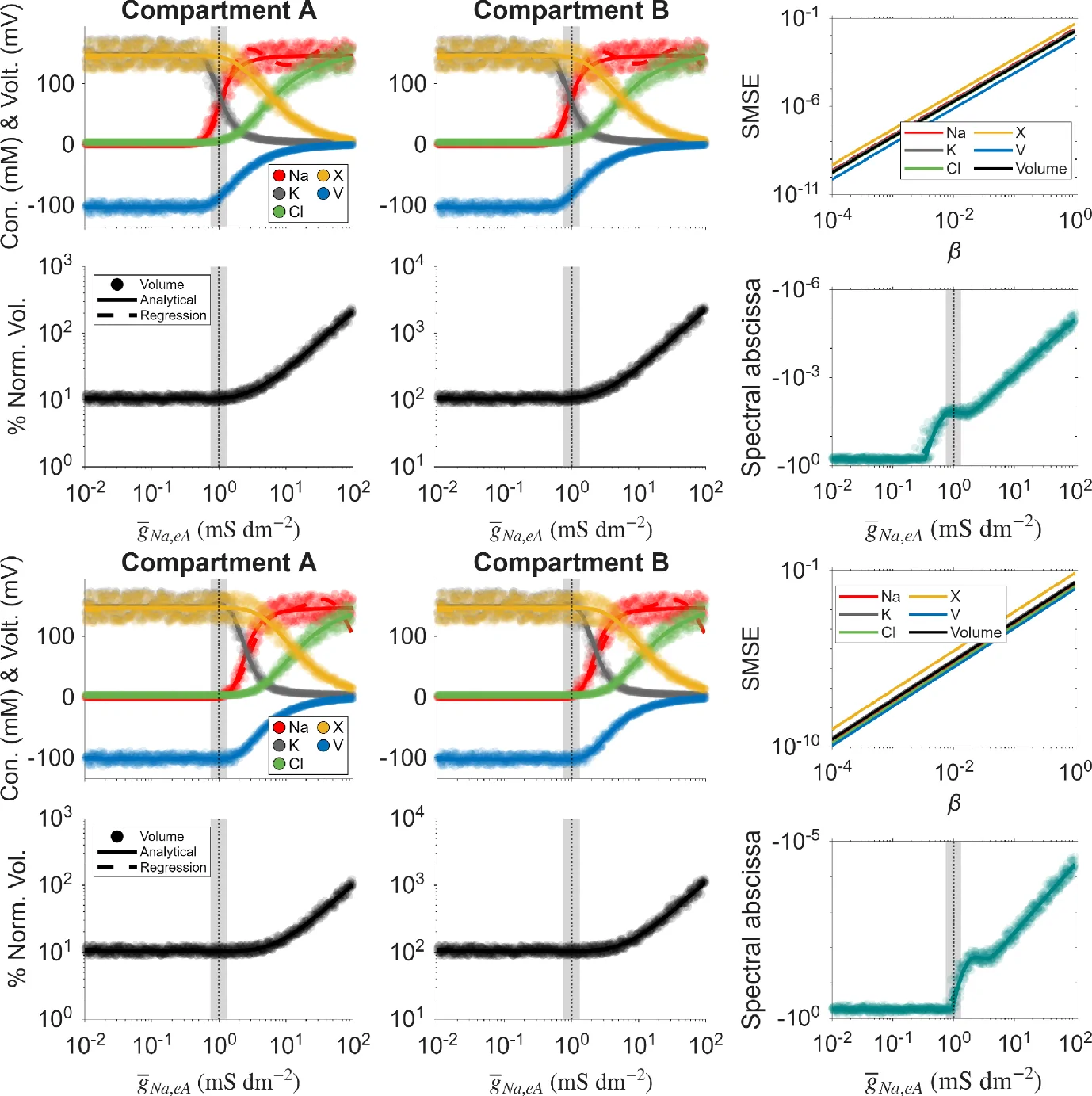
The steady states perturbed at p=40 (top) and p=90 (bottom) (color figure online)

**Fig. 24:**
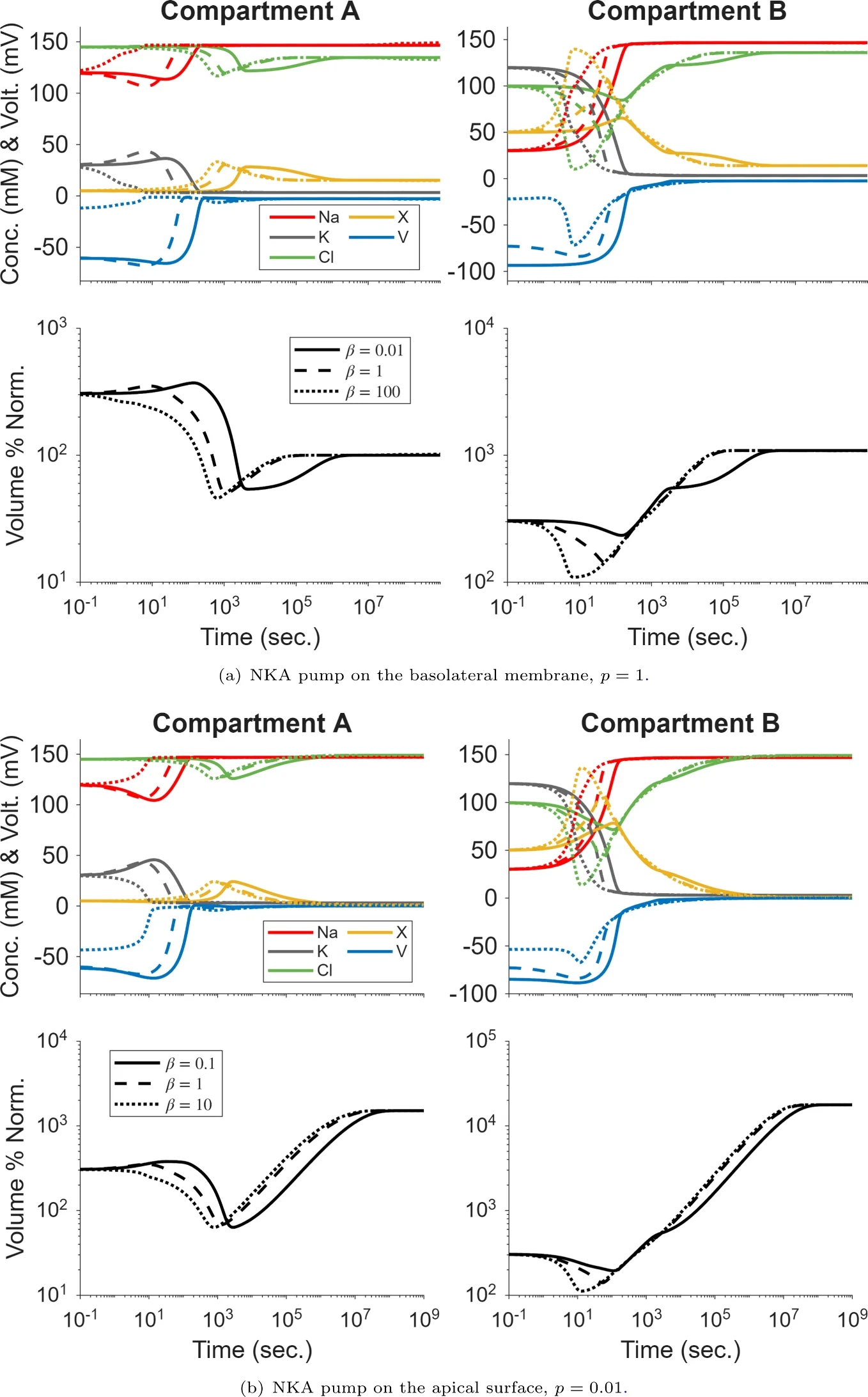
Time series of the ABp model with the NKA pump located on the basolateral membrane (a) and the apical surface (b), with all chloride conductances scaled as $\overline{g}_{Cl,(\cdot )}= \beta \times \overline{g}_{Cl,(\cdot )}^*$ for $\beta \in \{0.01, \, 1, \, 100\}$, shown by solid, dashed, and dotted lines, respectively. Although chloride conductances do not influence the steady-state values, the rate of convergence increases with increasing β (color figure online)

**Fig. 25:**
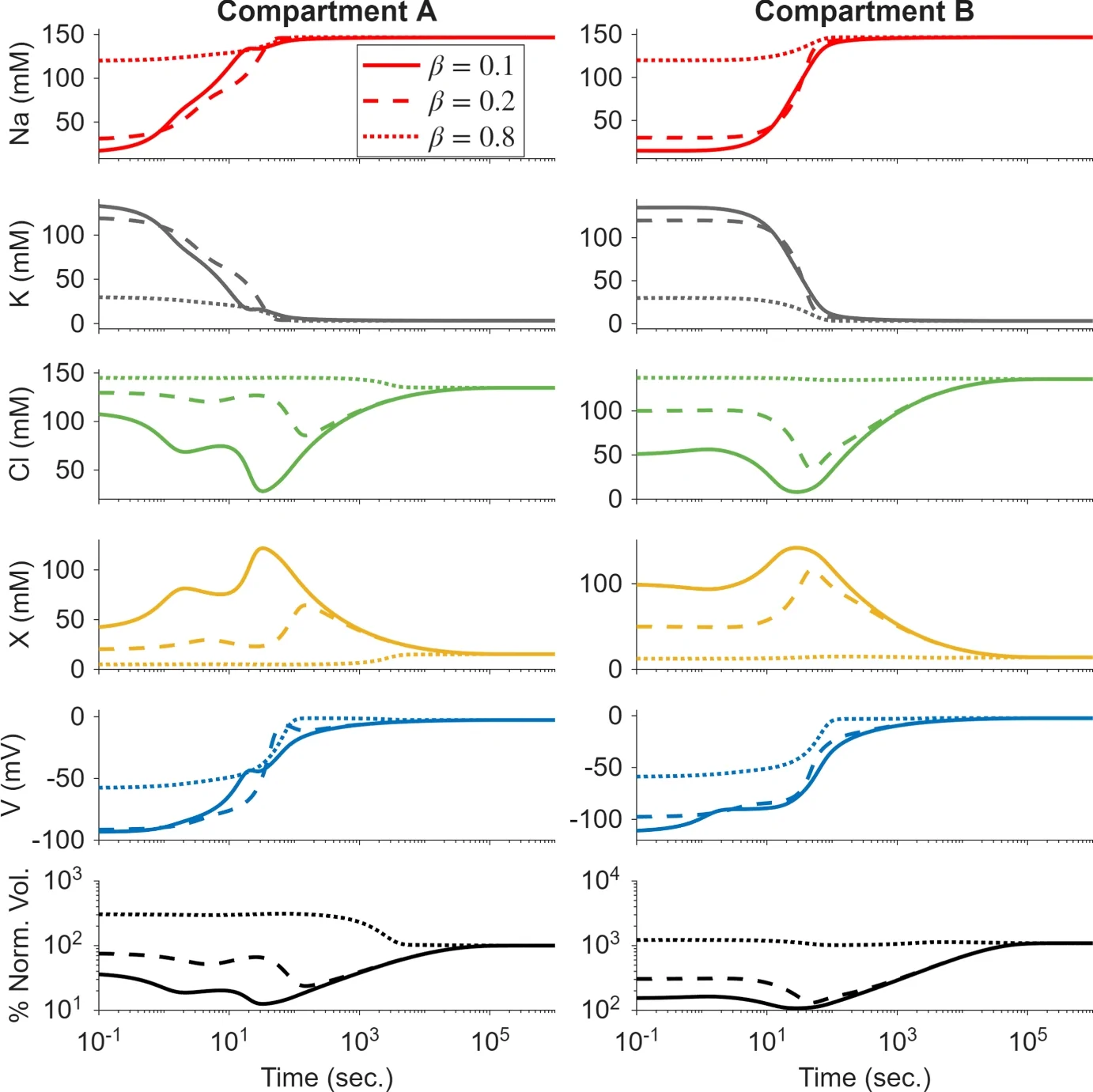
Time series of the ABp model with the NKA pump located on the basolateral membrane (p=1 μA,$\hbox {dm}^{-2}$) are shown for three different initial conditions parameterized by $\beta \in \{0.1,\, 0.2,\, 0.8\}$ (solid, dashed, and dotted lines, respectively). Results for compartment *A* and compartment *B* are displayed in the left and right columns, respectively. All trajectories converge to the same steady state without significant overshoot or undershoot (color figure online)

## A Appendix

### A.1 Default Values of the Coupled PLEs Model Parameters

In Tables 1–4, we introduce the parameters used in the coupled PLEs. We also provide *default* values for these parameters as a reference set. However, we emphasize that a main objective of this work is to avoid relying on a single parameter set and instead explore the system’s behavior over a wide range of parameter values. Note that the pump rate does not possess a default value; however, we are interested in compensatory mechanisms for when p is small, which is given here the value of p = 1 μA $\hbox {dm}^{-2}$.

**Table 1:** Parameters used in Equations (1b)–(4) and their default values

	Equations	Default value	Description
$a_{eA}$		$2\pi 10^{-7}$ $\text {dm}^2$	Basolateral membrane area
$a_{AB}$		$2\pi 10^{-7}$ $\text {dm}^2$	Apical surface area
$a_{eB}$		$2\pi 10^{-8}$ $\text {dm}^2$	Paracellular pathway area
$w_{A,0}$		$\frac{4}{3} \pi \cdot 125\cdot 10^{-15}$ $\hbox {dm}^{3}$	Initial volume of compartment *A*
$w_{B,0}$		$\frac{4}{3} \pi \cdot 125\cdot {10^{-15}}$ $\hbox {dm}^{3}$	Initial volume of compartment *B*
$X_A$	(7), (1b)	$5\cdot 10^{-3} \times w_{A,0}$ mol	Intra. impermeant molecules in *A*
$X_B$	(7), (1b)	$50\cdot 10^{-3} \times w_{B,0}$ mol	Intra. impermeant molecules in *B*
$z_{A}$	(7)	-1	Charge of impermeant molecules in *A*
$z_{B}$	(7)	-1	Charge of impermeant molecules in *B*
$C_{m,A}$	(3)	$10^{-4} { \, a_{eA}\text { F}}$	Total capacitance
$C_{m,B}$	(3)	$10^{-4} {\, a_{AB}\text { F}}$	Total capacitance
*R*	(4)	$8.314 \frac{\text {J}}{\text {mol K}}$	Universal gas constant
*F*	(3), (4)	$96485 \frac{{\text {C}}}{{\text {mol}}}$	Faraday constant
$\nu _{eA}$	(2a)	$126 \cdot 10^{-5} a_{eA}$ ${\text {dm}^6\cdot \text {mol}^{-1}\cdot \text {s}^{-1}}$	osmotic permeability × molar volume of water
			on basolateral membrane
$\nu _{AB}$	(2)	$126 \cdot 10^{-5} a_{AB}$ ${\text {dm}^6\cdot \text {mol}^{-1}\cdot \text {s}^{-1}}$	osmotic permeability × molar volume of water
			on apical surface
$\nu _{eB}$	(2b)	$126 \cdot 10^{-5} a_{eB}$ ${\text {dm}^6\cdot \text {mol}^{-1}\cdot \text {s}^{-1}}$	osmotic permeability × molar volume of water
			on para. pathway

**Table 2:** Parameters related to the extracellular space. Equations (7) and (40) are used to update $\mathcal {O}_e$ and $[Na]_e,[Cl]_e$, respectively

	Equation	Value	Description
$\mathcal {O}_e$	(1a), (8a), (2)	300 mM	Extra. osmolarity
$[Y]_e$	(7), (1a), (8)	1 mM	Extra. impermeant molecule
$z_{Y}$	(7), (8)	-1	Charge of extra. impermeant molecule
$[Na]_e$	(4), (7), (1a), (8b)	147 mM	Extra. Sodium concentration
$[K]_e$	(4), (7), (1a), (8b)	3 mM	Extra. Potassium concentration
$[Cl]_e$	(4), (7), (1a), (8)	149 mM	Extra. Chloride concentration
$[NaCl]_e$	(40)	0 mM	Extra. Salt concentration

**Table 3:** Default parameter values for temperature and conductances. Total conductance is defined as ${g}_{\text {ion},(\cdot )}=\overline{g}_{\text {ion},(\cdot )}\;a_{(\cdot )}$

	Equations	Value	Description
*T*	(4)	$37^{\circ }$C	Temperature
$\overline{g}_{Na,eA}$	(5a)	1 mS $\hbox {dm}^{-2}$	Na^+^ conductance on basolateral membrane
$\overline{g}_{Na,AB}$	(5a),(6a)	1 mS $\hbox {dm}^{-2}$	Na^+^ conductance on apical surface
$\overline{g}_{Na,eB}$	(6a)	1 mS $\hbox {dm}^{-2}$	Na^+^ conductance on para. pathway
$\overline{g}_{K,eA}$	(5b)	60 mS $\hbox {dm}^{-2}$	K^+^ conductance on basolateral membrane
$\overline{g}_{K,AB}$	(5b),(6b)	30 mS $\hbox {dm}^{-2}$	K^+^ conductance on the apical surface
$\overline{g}_{K,eB}$	(6b)	60 mS $\hbox {dm}^{-2}$	K^+^ conductance on para. pathway
$\overline{g}_{Cl,eA}$	(5c)	2 mS $\hbox {dm}^{-2}$	Cl^-^ conductance on basolateral membrane
$\overline{g}_{Cl,AB}$	(5c),(6c)	300 mS $\hbox {dm}^{-2}$	Cl^-^ conductance on apical surface
$\overline{g}_{Cl,eB}$	(6c)	10 mS $\hbox {dm}^{-2}$	Cl^-^ conductance on para. pathway

**Table 4:** Stoichiometry parameters

	Default value	Description
$\gamma _{Na}$	3	Sodium stoichiometry
$\gamma _{K}$	2	Potassium stoichiometry

### A.2 Motivation to Study a Larger Range for Each Parameter

Table 5 compares our default parameter values with those used in Weinstein (1992, 2012, 2015), and Edwards et al. (2014). We converted their permeabilities (reported as cm/sec) to conductances per unit area (mS/$\hbox {dm}^2$). We calculated conductance by differentiating the Goldman-Hodgkin-Katz (GHK) current-voltage equation with respect to voltage and evaluated it at the reversal potential. The reversal potentials were computed using the ion concentrations reported in each reference. The comparison for extracellular concentrations includes epithelial cells from the rat proximal tubule (Weinstein 1992, 2015), loop of Henle (Weinstein 2015), thick ascending limb and macula densa (Edwards et al. 2014), and a general biophysical representation of kidney function (Weinstein 2012).

**Table 5:** Comparison of selected parameter values reported in the literature

	Ours	(Weinstein 1992)	(Weinstein 2012)	(Weinstein 2015)	(Edwards et al. 2014)
$\overline{g}_{Na,eA}$ (mS $\hbox {dm}^{-2}$)	1	0.12497	113.30–12948.04	0.64–4.49	
$\overline{g}_{Na,AB}$ (mS $\hbox {dm}^{-2}$)	1	0		0.00–0.00	
$\overline{g}_{Na,eB}$ (mS $\hbox {dm}^{-2}$)	1			4165.83–8331.66	
$\overline{g}_{K,eA}$ (mS $\hbox {dm}^{-2}$)	60	24.608	79.65–5881.49	132.88–885.89	11.2262
$\overline{g}_{K,AB}$ (mS $\hbox {dm}^{-2}$)	30	110.736		16.61–110.74	1389.75
$\overline{g}_{K,eB}$ (mS $\hbox {dm}^{-2}$)	60			1784.08–3568.17	
$\overline{g}_{Cl,eA}$ (mS $\hbox {dm}^{-2}$)	2	0	93.31–24526.06	0.00–0.00	348.196
$\overline{g}_{Cl,AB}$ (mS $\hbox {dm}^{-2}$)	300	0		0.00–0.00	
$\overline{g}_{Cl,eB}$ (mS $\hbox {dm}^{-2}$)	10			1495.42–2990.84	
[Na$]_e$ (mM)	147	140	140		144
[K$]_e$ (mM)	3	4.9	4.9		5
[Cl$]_e$ (mM)	149	114	113.2		123

### A.3 Derivation of Voltage Expressions Under Electroneutrality Conditions

In Section 2, we derived the electroneutrality condition (7), i.e., for $j \in \{A,B\}$,

44 $$\begin{aligned} Q_j&= [Na]_j+ [K]_j-[Cl]_j+ z_j [X]_j= 0. \end{aligned}$$

Here, we use the electroneutrality condition to approximate the voltages in compartments *A* and *B*.

Multiplying this equation by $w_j$, differentiating it with respect to *t*, and using the right hand sides of (5)–(6), we obtain the following system for $V_A$ and $V_B$:

$$\begin{aligned} \begin{bmatrix}\alpha _1 & \alpha _2 \\ \alpha _2 & \alpha _3 \end{bmatrix} \begin{bmatrix}V_A\\ V_B\end{bmatrix} =\begin{bmatrix}\beta _1 \\ \beta _2 \end{bmatrix} \end{aligned}$$

where constant $\alpha _i$s and variable dependent $\beta _i$s are defined as below:

$$\begin{aligned}&\alpha _{1}=-(g_{Na,eA}+g_{Na,AB}+g_{K,eA}+g_{K,AB}+g_{Cl,eA}+g_{Cl,AB}) \\ &\alpha _2=g_{Na,AB}+g_{K,AB}+g_{Cl,AB}\\&\alpha _3=-(g_{Na,eB}+g_{Na,AB}+g_{K,eB}+g_{K,AB}+g_{Cl,eB}+g_{Cl,AB}) \\\\ \beta _1=&-(g_{Na,eA}+g_{Na,AB})E_{Na,A}- (g_{K,eA}+g_{K,AB})E_{K,A}- (g_{Cl,eA}+g_{Cl,AB})E_{Cl,A}\\&{+} g_{Na,AB}E_{Na,B}{+} g_{K,AB}E_{K,B}{+} g_{Cl,AB}E_{Cl,B}{+} (\gamma _{Na} {-} \gamma _{K})\,p({s}_{eA} a_{eA}+{s}_{AB} a_{AB}) \\ \beta _2=&-(g_{Na,eB}+g_{Na,AB})E_{Na,B}- (g_{K,eB}+g_{K,AB})E_{K,B}- (g_{Cl,eB}+g_{Cl,AB})E_{Cl,B}\\&+ g_{Na,AB}E_{Na,A}+ g_{K,AB}E_{K,A}+ g_{Cl,AB}E_{Cl,A}-(\gamma _{Na} - \gamma _{K})\,p{s}_{AB} \, a_{AB}\end{aligned}$$

The values ${s}_{eA}=1, {s}_{AB}=0$ and ${s}_{eA}=0, {s}_{AB}=1$ will be used for pump on the basolateral membrane and apical surface, respectively.

Solving the above linear system for $V_A, V_B$, we obtain:

45 $$\begin{aligned} V_A= \frac{\alpha _3 \beta _1 - \alpha _2 \beta _2}{\alpha _1 \alpha _3 - \alpha _2^2}, \quad \quad V_B= \frac{\alpha _1 \beta _2 - \alpha _2 \beta _1}{\alpha _1 \alpha _3 - \alpha _2^2}. \end{aligned}$$

### A.4 Sampling Procedure in VBGSA

In this work, we use variance-based global sensitivity analysis (VBGSA) to quantify simultaneous contribution of multiple model parameters to output variance. VBGSA computes Sobol indices, which decompose the total output variance into contributions from individual parameters and their interactions (Saltelli et al. 2004, 2008). A first-order Sobol index measures the fraction of output variance attributable to a single parameter alone, while higher-order indices capture joint effects.

As explained in the text, to apply VBGSA to the ABp model, we consider the interaction of nine parameters. Therefore, we consider a 9-dimensional parameter vector $\theta =(\theta _k)\in \mathbb {R}^9$ where the index k=1,…,9 refers respectively to $p,\; \overline{g}_{Na,eA},\; \overline{g}_{K,eA},\; \overline{g}_{Na,AB},\; \overline{g}_{K,AB},\; \overline{g}_{Na,eB},\; \overline{g}_{K,eB},\; T,\; [NaCl]_e,$ with default values denoted by $\theta _k^*$. Although we did not assign a default value to p (unlike the other parameters), because we are investigating compensatory parameters under reduced pump activity, we set a small pump rate value here, namely $\theta _{p}^* = 1\,\mu \text {A}\,\text {dm}^{-2}$.

For each parameter, we define a perturbation interval around its default value as

46a $$\begin{aligned} \Theta _k&:= \left[ \theta _k^*\,\left( 1-\beta \,\epsilon _k\right) ,\; \theta _k^* \,\left( 1-\beta \,\epsilon _k\right) ^{-1}\right] , \quad \quad \text{ for }\quad k\notin \left\{ T, \, [NaCl]_e\right\} , \end{aligned}$$

46b $$\begin{aligned} \Theta _k&:= \left[ \theta _k^*-\beta \,\epsilon _k,\; \theta _k^*+\beta \,\epsilon _k\right] , \hspace{2.6cm} \text{ for }\quad k\in \left\{ T, \, [NaCl]_e\right\} . \end{aligned}$$

Here, the scaling factor $\beta \in {[0,1]}$ serves as a uniform perturbation level across all nine parameters in the model, while the values of $\epsilon _k$ are fixed as

We then define the parameter hypercube, denoted by $\mathcal {H}^{9}$, as

47 $$\begin{aligned} \mathcal {H}^{9} \;:=\; \{ \theta \in \mathbb {R}^9 \, : \, \theta _k\in \Theta _k, \;\; \forall \, k\}. \end{aligned}$$

From the parameter hypercube $\mathcal {H}^{9}$, we draw $N = 10^6$ parameter points for VBGSA in Section 5.1 and $N = 10^3$ parameter points for robustness in Section 5.2. We use Latin hypercube sampling (LHS) to sample from $\mathcal {H}^9$.

The sampling procedure is as follows. Each marginal $\Theta _k$ is stratified into *N* equiprobable bins; one value is drawn without replacement from each bin. Independent random permutations are applied per parameter, and the draws are assembled to form *N* 9-dimensional samples (McKay et al. 1979; Helton et al. 2006). To sample from $\mathcal {H}^9$, LHS is implemented as follows. For each *k*, write $\Theta _k=[a_k,b_k]$ and define the monotone transform

$$ \phi _k(t)= {\left\{ \begin{array}{ll} \log t, & k\notin \{T,[NaCl]_e\},\\ t, & k\in \{T,[NaCl]_e\}, \end{array}\right. } \qquad t\in \Theta _k, $$

with inverse $\phi _k^{-1}(y)=\exp (y)$ for the log case and $\phi _k^{-1}(y)=y$ otherwise. Partition [0, 1] into *N* strata $I_i=\bigl ((i-1)/N,i/N\bigr ]$. For each parameter *k*, draw $U_{i,k}\sim \textrm{Unif}(I_i)$ independently across *i*, apply an independent random permutation $\pi _k$ of $\{1,\dots ,N\}$, and set for i=1,⋯,N $\theta _k^{(i)} =\phi _k^{-1}\!\Big (\phi _k(a_k) + U_{\pi _k(i),k}\,[\,\phi _k(b_k)-\phi _k(a_k)\,]\Big ).$ The *N* vectors $\theta ^{(i)}=(\theta ^{(i)}_{{p}},\dots ,\theta ^{(i)}_{{[NaCl]_e}})$ form Latin hypercube sampling on $\mathcal {H}^9$ with uniform marginals on $\Theta _k$ for $k\in \{T,[NaCl]_e\}$ and uniform marginals on $\log \Theta _k$ for $k\notin \{T,[NaCl]_e\}$ (McKay et al. 1979; Helton et al. 2006; Dela et al. 2022). Positivity of the log-scaled bounds is guaranteed by $0<\beta \,\epsilon _k\ll 1$ in (46a). For LHS sampling in VBGSA, β is fixed at β=1.

In the figures, we plot responses versus the predefined $\Theta _k$ domain for the parameter indexed by *k*. When helpful for visualization, the domain $\Theta _k$ for the parameter of interest may be extended beyond $\Theta _k$, while for the remaining eight parameters the sampling domain $\mathcal {H}^9$ in (47) remains unchanged. In the same plots against parameter *k* we will highlight the original domain $\Theta _k$ with a light gray vertical band centered around $\theta _k^*$. Unless otherwise noted, we set β=1.

### A.5 Heat Map Analysis of Key Parameters

In Section 5.1, we presented a heat map for p∈[0.1,150] (in μA,$\hbox {dm}^{-2}$) and basolateral sodium conductance $\overline{g}_{Na,eA}\in [0.01, 100]$ (in mS,$\hbox {dm}^{-2}$). Here, we perform an analogous analysis by varying the pump rate together with the apical sodium conductance, i.e., p∈[0.1,150] and $\overline{g}_{Na,AB}\in [0.01, 100]$, with results shown in Figure 19. We repeat this procedure for the alternative paracellular sodium conductance parameter, presenting the corresponding heat map in Figure 20. As explained in the text, for a fixed small p, indicated by black vertical line, decreasing $\overline{g}_{Na,AB}$ (respectively, $\overline{g}_{Na,eB}$) slightly decreases the volume of *A* but substantially increases (respectively, decreases) the volume of *B*, as indicated by the Sobol indices, Figure 8. All panels in these heat maps use the same layout as those in Figure 9.

Changes in extracellular sodium and chloride concentrations alter the osmotic balance across the membrane and drive water influx or efflux, a fundamental principle of cell volume regulation (Hoffmann et al. 2009). While extracellular ion concentrations are tightly regulated *in vivo*, such perturbations can be imposed experimentally or clinically, for example through controlled changes in perfusate composition or osmotherapy. This principle underlies clinical interventions such as hypertonic saline treatment for cerebral edema (Simard et al. 2007).

As indicated by the Sobol index diagram in Figure 8, $[NaCl]_e$ and *T* also influence the steady-state volumes of compartments A and B. In what follows, we vary the pump rate together with each of these parameters over broader perturbation ranges than those used in Figure 8 to examine their combined effects.

Figure 21, where p∈[0.1,150] and $[NaCl]_e\in [-50,\,50]$, shows that increasing $[NaCl]_e$ leads to a decrease in the steady-state cell volume, especially when the pump rate is low, as indicated by the vertical black arrow in the right panel. In addition, the figure shows that intracellular sodium and chloride concentrations increase monotonically as the extracellular sodium-chloride concentration increases. Due to the balanced charges carried by sodium and chloride, the model predicts that the membrane voltage remains unaffected by changes in extracellular sodium-chloride concentration. The intracellular potassium concentration remains constant in order to maintain electroneutrality and positive osmolarity.

In an *ex vivo* study, rabbit proximal tubule cells—specialized epithelial cells in the kidney nephron—were observed to shrink and remain at a reduced volume following the abrupt addition of NaCl to the bathing solution. In contrast, partial volume recovery occurred when an equivalent osmotic challenge was applied using mannitol, a metabolically inert solute commonly employed as an osmotic control (Gagnon et al. 1982). In a separate *ex vivo* study, rabbit proximal tubule cells were able to maintain nearly constant volume when extracellular osmolality was increased gradually through the addition of NaCl to the bath (Lohr et al. 1989). Within this experimentally controlled context, our theoretical results predict that modulation of extracellular $[Na]_e$ and $[Cl]_e$ provides an effective means to influence cell and lumen volume.

Finally, we plot the steady states as functions of p and *T* in Figure 22. To probe the model’s sensitivity, we sweep a wide temperature range (); biological interpretation is restricted to temperatures where the system remains aqueous and active transport is meaningful. Our model predicts that, for a fixed p, lowering the temperature leaves the voltage and intracellular sodium and potassium concentrations essentially unchanged. We observe a modest decrease in intracellular chloride and in cell volume as *T* decreases. As shown in the right panel of Figure 22, the effect on $w_A^{ss}$ is most pronounced when p is small (see the vertical arrow). For larger values of p, temperature has only a minor effect on $w_A^{ss}$. The small magnitude of this response over a physiologically reasonable range suggests that temperature modulation may serve as only a modest volume-directed intervention when NKA activity is limited. Similar trends hold for $w_B^{ss}$ (not shown).

*In vitro* recordings from rat type IIb omohyoid skeletal muscle fibers showed that, at $19^{\circ }$C, voltages did not significantly differ from voltages at $37^{\circ }$C or any other tested temperature in between (Ruff 1999). Preoptic-anterior hypothalamic neurons measured between 30 and $40^{\circ }$C showed no significant differences in their resting potential (Zhao and Boulant 2005). Direct mammalian, isomotic steady state evidence for cooling-induced cellular shrinkage is sparse; however, related settings report smaller cellular water content at lower temperatures. For instance, rat hepatocytes shrink during hypothermic storage, particularly when colloid support is absent (Neveux et al. 1997), and mild hypothermia reduces cytotoxic cellular swelling in brain tissue (Schwab et al. 1998).

### A.6 Robustness Analysis for Intermediate and High Pump Rates

The robustness analysis on $\overline{g}_{Na,eA}$ is repeated for p=40 and $90 \; \mu \text {A dm}^{-2}$. The overall trends are the same as Figure 11 when $p=1\;\mu \text {A dm}^{-2}$ – increasing $\overline{g}_{Na,eA}$ will increase $[Na]_j$, $[Cl]_j$, $V_j$, and $w_j$ while decreasing $[K]_j$ and $[X]_j$. Compared to the case in Figure 11, there is a difference in the values of the steady state and spectral abscissa. For the intermediate and high pump rate, the $[Na]_j^{ss}$ and $[Cl]_j^{ss}$ will increase from near 0 mM to about 150 mM. $V_j^{ss}$ increases from roughly -100 mV to near 0 mV. $[X]_j^{ss}$ and $[K]_j^{ss}$ decrease from roughly 150 mM to about 0 mM. $w_A^{ss}$ increases from $\sim 10 \%$ to $\sim 100 \%$, and $w_B^{ss}$ increases from $\sim 100 \%$ to ∼1100 (volumes are normalized by $w_{\text {Norm}}$). Notice that the trends observed here are the same as those seen in Figure 9. The spectral abscissa increases from -1 to $-10^{-5}$. At intermediate and high pump rates, some default steady-state values (e.g., $[Na]_j^{ss}$) approach zero, causing the denominator in RMSRE to become singular. We therefore replace RMSRE with the standardized mean square error (SMSE) (Rasmussen and Williams 2006), which normalizes by the variance of the perturbed outputs rather than by the default value and remains well-defined when the reference is near zero. SMSE is defined as $\text {SMSE}=\frac{\mathbb {E}\!\left[ ({\boldsymbol{y}}-{\boldsymbol{o}})^2\right] }{\operatorname {Var}({\boldsymbol{o}})}.$ These two cases are summarized in Figure 23.

### A.7 Time-Series Simulations of ABp Model

Although the main focus of this work is the analysis of stationary solutions of the ABp model, in this section we present numerical simulations and corresponding time series to support some of our claims and to illustrate predictions that will be explored in future work.

**Effect of chloride conductances.** As discussed in the main text, the steady-state solutions of the ABp model and the sign of the corresponding eigenvalues do not depend on the chloride conductances. However, the magnitude of the eigenvalues—and hence the rate of convergence to the steady state—does depend on the chloride conductances. In particular, in Equations (5c)–(6c), scaling all chloride conductances by a factor much less than 1 (i.e., multiplying the right-hand side by a small factor) is expected to slow convergence to the steady state.

To visualize this effect, we simulate the ABp model for different values of the chloride conductances. Figure 24 depicts time series for multiple values of the chloride conductances when the pump is located on the basolateral membrane (Figure 24(a)) and on the apical surface (Figure 24(b)). As shown in the figure, the steady-state values remain unchanged, while the rate of convergence to the steady state increases as the chloride conductances are scaled (by a factor β), confirming our theoretical results. All other parameters are held fixed, and the initial condition is given by

$$\begin{aligned}&[Na]_A(0)= 120, \; [K]_A(0)= 30, \; [Cl]_A(0)=145 \;\;\text {mM}, \nonumber \\&[Na]_B(0)=30, \; [K]_B(0)=120,\; [Cl]_B(0)=100\;\;\text {mM}. \end{aligned}$$

The initial conditions for the volumes are the same as those specified in Table 1.

**Transient response to different initial conditions.** In the main text, we proved that, under certain conditions, the ABp model admits a unique and locally stable steady state. However, this result does not preclude the possibility of large transient excursions that could drive concentrations or volumes to physiologically extreme values before settling. Here, for the ABp model with parameters set to their default values, we plot several solutions starting from different initial conditions in Figure 25. Note that electroneutrality is imposed on the initial conditions. We observe that the solutions do not exhibit significant over- or undershoot before converging to their steady states. Although this observation is limited to a single parameter set and a few initial conditions, it suggests a potential direction for future work, namely, establishing boundedness properties of the ABp model solutions.
